# Costs of clinical trials with anticancer biological agents in an Oncologic Italian Cancer Center using the activity-based costing methodology

**DOI:** 10.1371/journal.pone.0210330

**Published:** 2019-01-08

**Authors:** Giacomo Pascarella, Arturo Capasso, Antonio Nardone, Maria Triassi, Sandro Pignata, Laura Arenare, Paolo Ascierto, Marcello Curvietto, Piera Maiolino, Roberta D’Aniello, Agnese Montanino, Francesca Laudato, Gianfranco De Feo, Gerardo Botti, Francesco Perrone, Antonella Petrillo, Ernesta Cavalcanti, Secondo Lastoria, Nicola Maurea, Alessandro Morabito

**Affiliations:** 1 Scientific Directorate, Istituto Nazionale Tumori "Fondazione G. Pascale", IRCCS, Napoli, Italy; 2 Dipartimento di Diritto, Economia, Management e Metodi Quantitativi, Università degli Studi del Sannio, Benevento, Italy; 3 Dipartimento Sanità Pubblica, Università Federico II, Napoli, Italy; 4 Dipartimento di Oncologia Uro-Ginecologica, Istituto Nazionale Tumori, “Fondazione G.Pascale”, IRCCS, Napoli, Italy; 5 Melanoma, Cancer Immunotherapy and Innovative Therapies Unit, Istituto Nazionale Tumori,“Fondazione G.Pascale”, IRCCS, Napoli, Italy; 6 Hospital Pharmacy, Istituto Nazionale Tumori, “Fondazione G.Pascale”, IRCCS, Napoli, Italy; 7 Thoracic Medical Oncology, Istituto Nazionale Tumori, “Fondazione G.Pascale”, IRCCS, Napoli, Italy; 8 Clinical Trials Unit, Istituto Nazionale Tumori, “Fondazione G.Pascale”, IRCCS, Napoli, Italy; 9 Radiology Unit, Istituto Nazionale Tumori, “Fondazione G.Pascale”, IRCCS, Napoli, Italy; 10 Laboratory Medicine Unit, Istituto Nazionale Tumori, “Fondazione G.Pascale”, IRCCS, Napoli, Italy; 11 Nuclear Medicine Unit, Istituto Nazionale Tumori, “Fondazione G.Pascale”, IRCCS, Napoli, Italy; 12 Division of Cardiology, Istituto Nazionale Tumori, “Fondazione G.Pascale”, IRCCS, Napoli, Italy; Universitatsspital Basel, SWITZERLAND

## Abstract

**Aim:**

The aim of the present study was to assess the estimated “per patient” total cost for a single Oncologic Italian Cancer Center participating in a multicenter clinical trial with new anticancer biological agents using the activity-based costing (ABC) methodology.

**Methodology:**

Nine randomized phase 3 clinical trials employing biological agents at the National Cancer Institute of Napoli, Italy, were analyzed to indentify “per patient” costs of each trial, according to the ABC methodology. The average consumption of resources for a patient completing the entire planned treatment was estimated for each trial. Through interviews of the personnel (doctors, nurses and technicians) and by analyses of the clinical trials protocols, the main activities of the 9 clinical trials were identified and, for each trial, the complete health care pathway of the patients and the treatment programmes were minutely reconstructed. Drug costs were not included because provided by Sponsors.

**Principal findings:**

The average costs of the pre-study, treatment, monitoring, follow-up, audit, and administrative activities accounted for 2.357, 4.783, 700, 372, 1.263, and 9 Euro, respectively. The average total cost estimated for all “per patient” activities, including overhead costs, was 11.379 Euro. Staff costs accounted for € 5.988, while costs of diagnostic test accounted for 3.494 Euro. Clinical trials with immunotherapeutic drugs accounted for higher costs (+601 Euro as oncological staff costs, +1.318 Euro as intermediate services cost and +384 Euro as overheads).

**Conclusions:**

The average total cost estimated for all “per patient” activities of a clinical trial with new anticancer biological agents was 11.379 Euro using the ABC methodology.

## Introduction

Clinical research plays a critical role for drugs development. However, cost of clinical trials for a participating clinical centre is currently unknown and difficult to determine [1]. Revenues are quite easy to measure, as they are related to the activities specified in the clinical trial protocol, but the same cannot be said for costs.

According to the traditional managerial accounting systems based on cost centers, the costs typically related to the activities of clinical trials are mostly indirect (staff costs, diagnostic test costs, medical instruments maintenance and depreciation costs, etc), as it is not possible to assess the amount of any specific resource used for each trial through objective measure. Therefore, all production costs are charged directly to the center where the consumption of the resources takes place and only indirectly to the patients receiving the treatment.

In this context, the activity-based costing (ABC) methodology can be used to overcome the shortfalls of the traditional cost accounting systems, especially when indirect costs, not directly traceable to the products-services, represent an important proportion of the total cost [2]. ABC traces the resources to activities to facilitate costing of products and services. It assumes that activities consume resources and products consume activities. Therefore, indirect production costs are first assigned to the activities (through appropriate resources driver), determining the costs of the identified activities. Then, the activities costs are allocated to products or services, according to the activities consumed by each of them, using activity measures and activity rates [3]. Accordingly, ABC allows to determine the standard full cost per service unit provided by the hospital (for instance, the unit patient cost), as a tool for administrative cost information, strategic decision-making, quality and efficiency improvement [3].

The aim of the present study was to assess the estimated “per patient” total cost for a single Oncologic Italian Cancer Center participating in a multicenter clinical trial with new anticancer biological agents using the ABC methodology, with the purpose to develop a model capable of providing accurate and relevant economic information for a “profit” clinical trial.

## Methods

ABC in health care organization follows the same typical steps of ABC systems implemented in a manufacturing company [4–6]. It includes: setting the key variables (cost objects, main activities and resources used); defining a process-based map representing the flow of the activities, resources and their interrelationships; allocating resources cost to activity pool; attributions of secondary activities cost to primary activities, assessing activity consume by each cost objects; computing activity rate; sharing activities cost to products that caused that activity [7].

The cost objects have been identified in the patients participating into profit randomized clinical trials with either immunotherapeutic or target based agents. Therefore, we have analyzed nine randomized phase 3 clinical trials with new anticancer biological agents carried out at the Thoracic, Melanoma, and Uro-Gynecological Medical Oncology of the National Cancer Institute of Napoli, Italy in order to indentify “per patient” costs. For each trial we considered an “ideal patient” representing the consumption of resources for completing the entire planned treatment. We used semi-structured interviews with the principal investigators and key personnel (doctors, nurses and technicians) to identify the primary activities required by the patients of 9 clinical trials, reported in S1 Table and S1 Text. All employees provided their oral informed consent to have their interviews used in the study.

**Fig 1:**
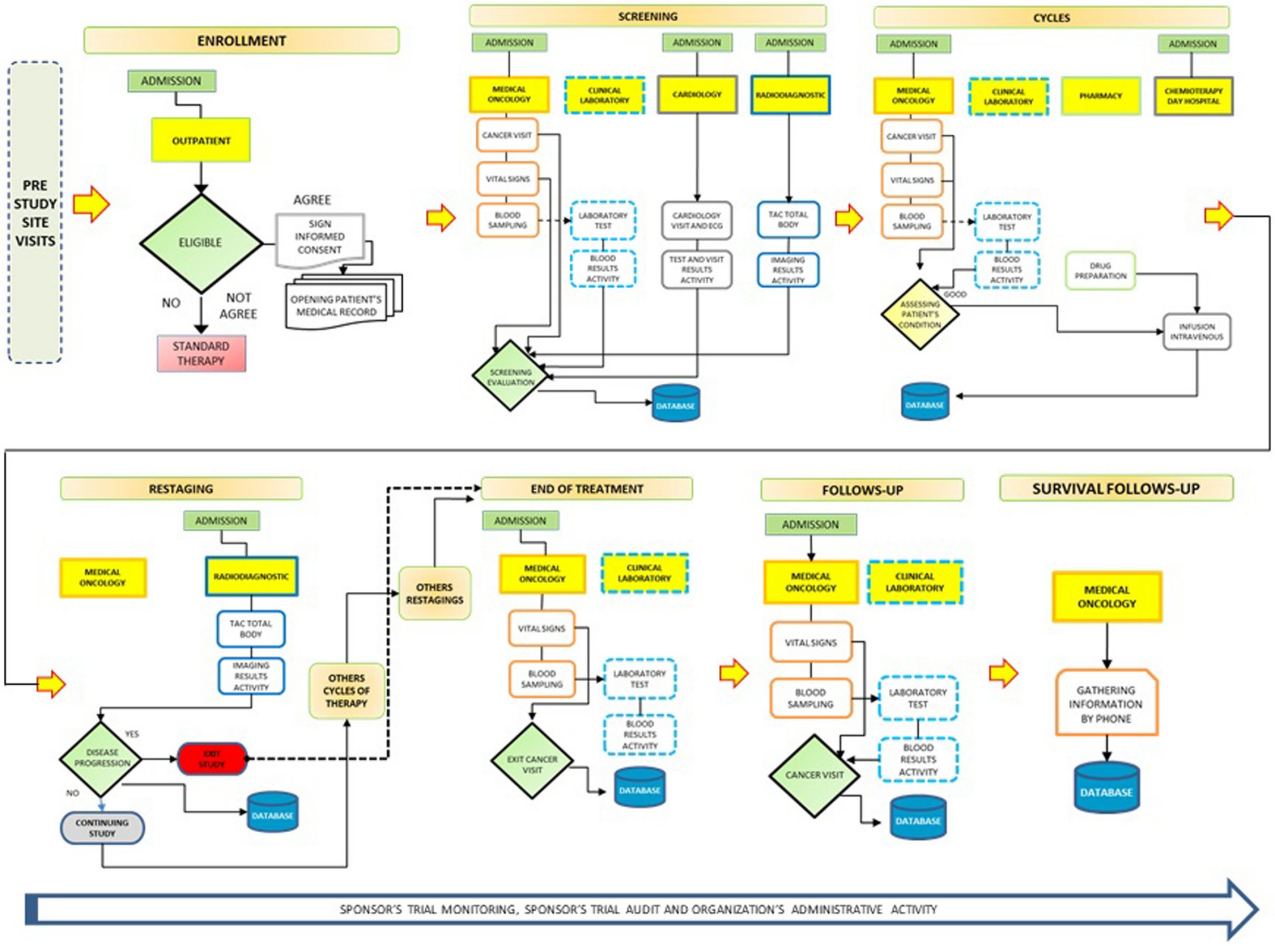
Patient clinical pathway of a clinical study.

Through key personnel interviews and by analyses of the clinical trials protocols, it was minutely reconstructed the whole patient hospital pathway and the treatment schedule, the time required to perform all the planned activities, the name and the role of the employees who performed them (e.g., doctors, nurses, pharmacists, laboratory technicians, x-ray technicians, study coordinators, etc.) for all unit involved in the studies (Fig 1).

About resources’ cost used, we have considered only two main cost categories, those related to staff involved and diagnostic tests performed in the clinical study, because we didn’t have already defined costs provided by cost center accounting system. The staff’s cost was estimated multiplicand the company hourly labour cost of each employee for the time required to perform a certain activity, both express in minutes, while the cost of the diagnostic tests and other procedures was estimated by using public local health reimbursement [8]. All other costs, not directly related to a specific activity, were included in an overhead cost category estimated at 20% of the cost of all activities performed, comprehensive of costs connected with infrastructures and the general operation of the organisation, including hiring or depreciation of buildings and plants, water/gas/electricity, maintenances, insurances, supplies and petty office equipments, communication and connection costs, postage, and costs connected with horizontal services such as administrative and financial management, human resources, training, legal advice, documentation, etc. Finally, drug costs were not included because provided by the sponsor.

We built an Expense-Activity-Link Matrix (EAL-matrix) for each clinical trial examined as illustrated in S2 Table. The EAL-matrix reflects the first stage of cost assignment and represents a first map of interrelations between activities performed and resources consumed. In the first column we find the main activities performed grouped into activity pools, in the middle columns the Units involved in clinical trials and the type of resource consumed by activities, differentiated in staffs and diagnostics tests costs. The last column on the right shows the cost of every activity pool obtained by adding the corresponding value of the resources consumed. So we used the time spent on each activity as resource driver to allocate the staff costs and the number of tests by type required to drive the cost of the diagnostic tests, while their monetary valuation was done using hourly labour cost and through the system of healthcare reimbursement, respectively. Since we hypothesized the enrollment of a single patient type, the last column of EAL-Matrix represents also the activities demand value made by patient. Therefore, our patient type has alone requested and consumed all the activities identified, plus an amount of consumption of intermediate and facility services, that we estimated equal to 20% of all activities’ cost demanded.

Since the clinical trials were structured differently, in order to facilitate also data representation, a bridging table was developed by grouping all the primary activities performed in six main categories, as follows: a) pre-study activities, including “Pre-Study Site Visit, Enrollment and Screening Phases”; b) treatment, including “Cycles, Restaging and Exit Cancer Visit”; c) trial monitoring; d) follow-up; e) audit (generally only one for each study); f) administrative activities. Therefore, we summarized all data related to the 9 clinical trials in a new EAL-matrix (S3 Table). Like the EAL-matrix, also the new EAL-Matrix shows the main activities performed (rows), and the organizational units involved in patient’s care process to perform diagnostic tests required by clinical trial protocol (column).

## Results

The characteristics of the 9 clinical trials carried out with anticancer biological agents from 2014 to 2016 at the National Cancer Institute of Napoli Italy are reported in Table 1. They were all randomized clinical trials with immunotherapeutic or target based agents for melanoma, lung and ovarian cancers. The results for each clinical trial are summarized in S4–S6 Tables, while details in term of resources consumed, differentiate in staff and test costs, are specified in S7–S9 Tables. Using EAL-Matrix setting, in S10–12 Tables, we have provided a detail of staff involved in each study and replaced the costs with the time spent in minutes. We then grouped the same data for staff roles in order to facilitate the overall calculation of the time spent by each operator on the activities identified. S13 Table reports, instead, the minimum and maximum hourly rates differentiated for staff role, as well as the minimum and maximum hourly rates for staff role for each clinical study. Some differences in hourly cost for the same staff role are related to general employment contract provisions (i.e., project contract rather than permanent contract work). Finally, S14 Table shows the diagnostic tests’ type and cost for each clinical study (in original language) and S15 Table is an example of collected data of a clinical trial used to estimate the”per patient” total cost. In Table 2 are reported, instead, the estimated cost of the primary activities, the overall cost of the activities performed by the single units involved in the study and, finally, the estimated “per patient” total cost of all nine studies.

**Table 1:** Characteristics of the trials.

Type of cancer	Drug class	Investigational drug	Setting
Ovarian	PARP inhibitor	Rucaparib	Maintenance therapy
Urothelial	Anti PD-1 inhibitor	Pembrolizumab	Second line therapy
Ovarian	PARP inhibitor	Olaparib	Maintenance therapy
Lung	Anti PD-1 inhibitor	Nivolumab	First line therapy
Lung	Anti EGFR tyrosine kinase inhibitor	Dacomitinib	3–4 line therapy
Lung	Anti HER 3 monoclonal antibody	Patritumab	2–3 line therapy
Melanoma	Anti PD-1 inhibitor	Nivolumab	2 line therapy
Melanoma	Anti PD-1 inhibitor	Pembrolizumab	2 line therapy
Melanoma	BRAF inhibitor	Vemurafenib	Pretreated or not

**Table 2:** EAL matrix (average costs “per patients” of all studies).

ACTIVITY POOL	MEDICAL ONCOLOGIES	RADIODIOLOGY	NUCLEAR MEDICINE	PHARMACY	CLINICAL LABORATORY	PATHOLOGY	CARDIOLOGY	MOLECULAR BIOLOGY	ACTIVITY COST POOL
PRE-STUDY ACTIVITIES	643,37	837,08	29,90	89,51	490,82	38,57	211,80	15,71	2.356,75
TREATMENT	552,00	1.934,02	106,80	516,21	1.596,32	0,00	59,73	17,72	4.782,80
TRIAL MONITORING	699,72	0,00	0,00	0,00	0,00	0,00	0,00	0,00	699,72
FOLLOW-UP	115,07	0,00	0,00	0,00	256,91	0,00	0,00	0,00	371,98
AUDIT	804,71	130,00	8,40	63,28	25,30	0,00	225,91	5,32	1.262,92
ADMINISTRATIVE ACTIVITY	8,68	0,00	0,00	0,00	0,00	0,00	0,00	0,00	8,68
**ESTIMATED** **TOTAL COST**	**2.823,55**	**2.901,09**	**145,09**	**669,00**	**2.369,36**	**38,57**	**497,44**	**38,75**	**9.482,85**
OVERHEAD COST (20%)									1.896,57
**ESTIMATED TRIAL TOTAL COST**									**11.379,42**

The average costs of pre-study, treatment, monitoring, follow-up, audit, and administrative activities accounted for 2.357, 4.783, 700, 372, 1.263, and 9 Euro, respectively. The average total cost estimated for all activities accounted for 9.484 Euro. Overall, the average estimated “per patient” cost, including the overhead costs, accounted for 11.379 Euro.

**Fig 2:**
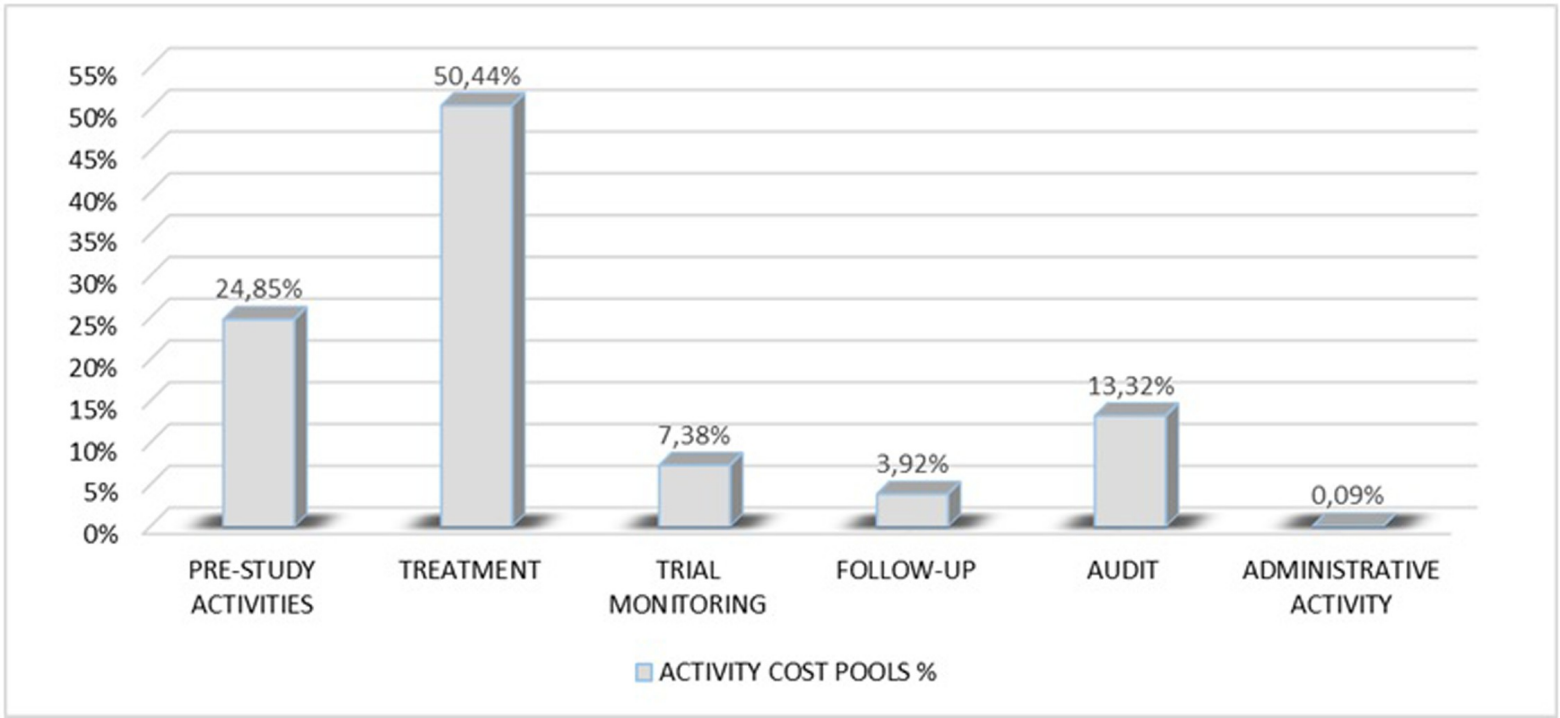
Incidence rates of activity cost pool.

The activities related to patients’ treatment take up, still in average, about the 50,44% of the resources’ cost used (excluding the overhead costs share). Pre-study, trial monitoring and audit activities roughly represented 46%, while follow-up and administrative activities approximately 3,92% and 0,09% respectively (Fig 2).

**Fig 3:**
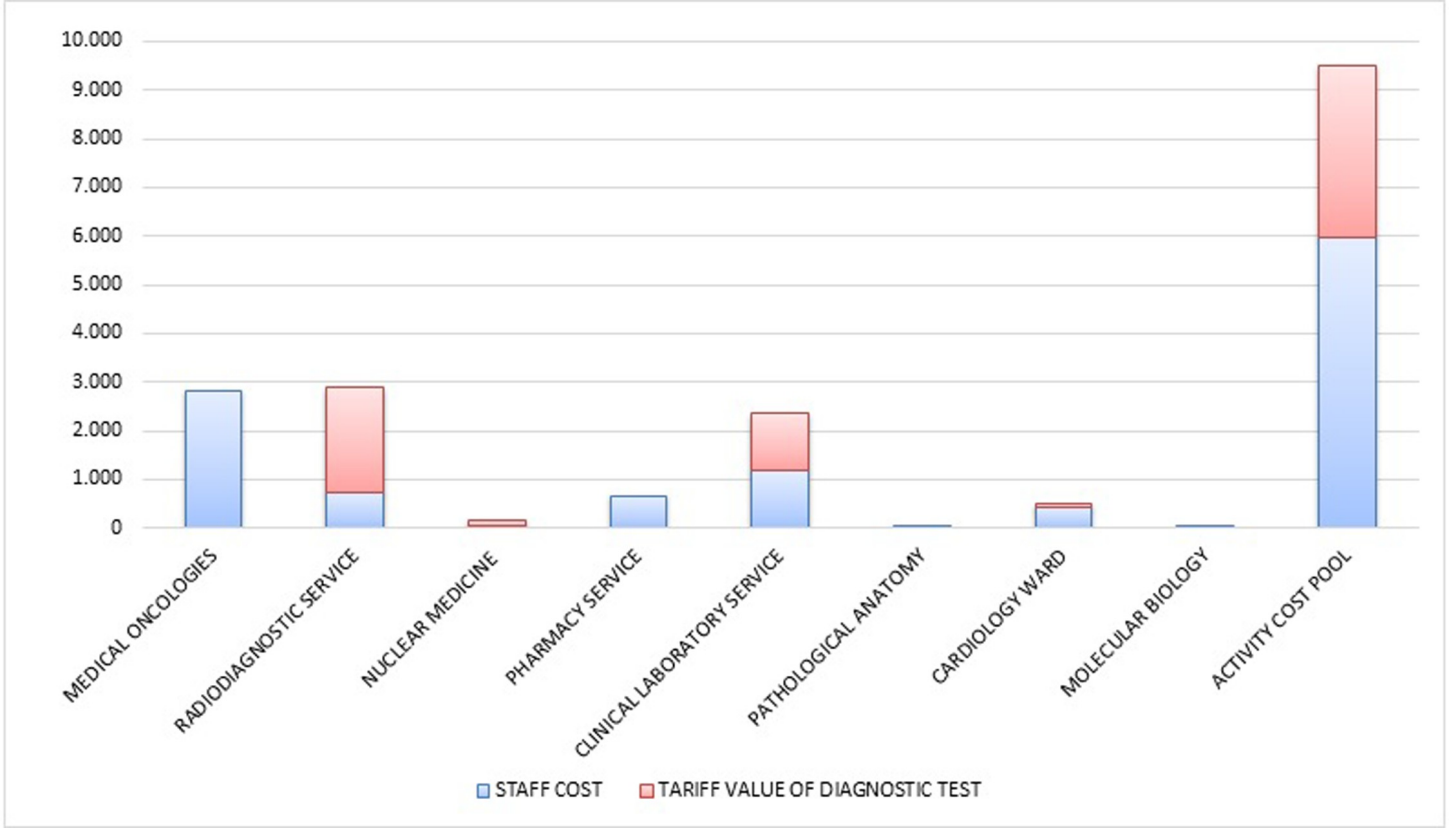
Cost composition separate for organizational unit involved (average values).

**Fig 4:**
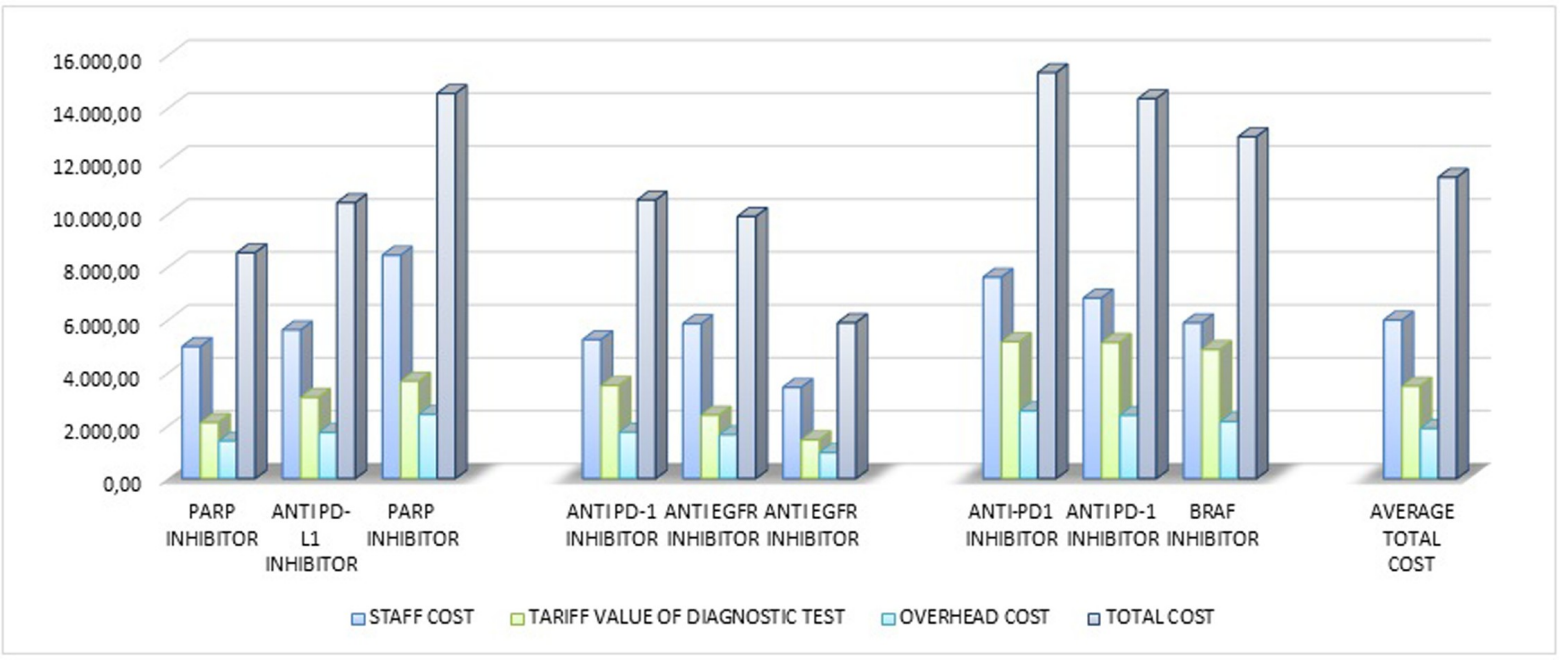
Cost composition separate for clinical trial.

Fig 3 shows the estimated cost of resources in its two main components (staff costs and diagnostic test costs), grouped on the Units involved in patients care. In average, staff costs accounted for 5.988 Euro, while costs of diagnostic test accounted for 3.494 Euro. Overall, staff costs account for about 63% of the estimated activity cost pool, without overhead costs. The total costs of each clinical trial, grouped in three main categories, including staff, diagnostic test and overhead costs are reported in Fig 4. Considering also overhead costs share, staff and diagnostic costs both decrease from 63% to 52% and from 37% to 30% respectively.

**Fig 5:**
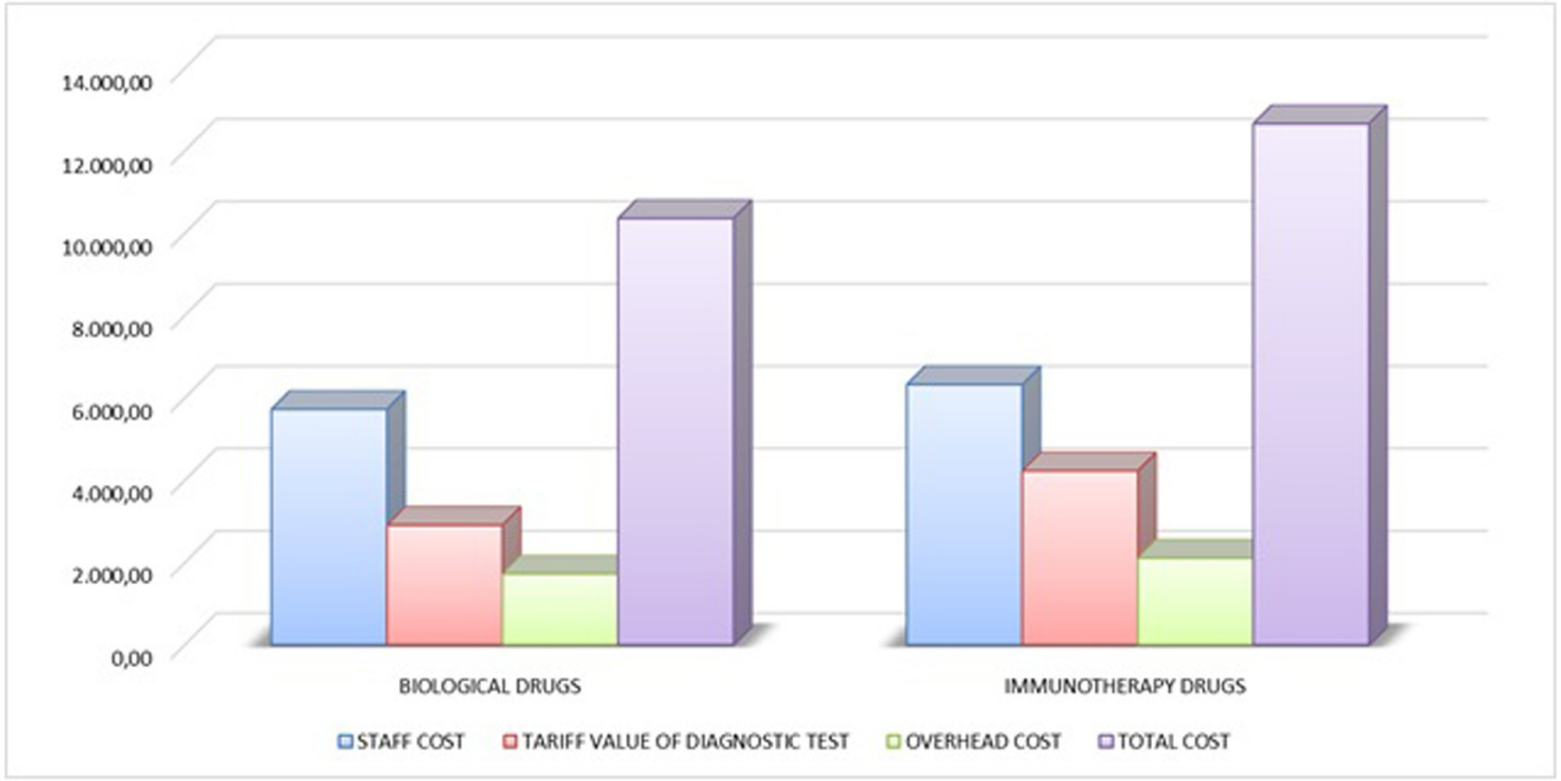
Cost composition separate for the type of the experimental drug (average value).

The average cost of the nine studies grouped according to the typology of the experimental drug (5 studies with target based agents and 4 studies with immunotherapeutic drugs) are reported in Fig 5. The collected data indicate that the clinical trials with immunotherapeutic drugs accounted for higher costs (+601 Euro as oncologic staff costs, +1.318 Euro as intermediate services costs and +384 Euro as overhead costs). In average, a trial with immunotherapy drugs accounts for 12.659 Euro per patient, namely 2.302 Euro more than a clinical trial with target based agents.

## Discussion

This study tried to define the estimated “per patient” total cost for a single Oncologic Italian Cancer Center participating in a multicenter clinical trial with new anticancer biologic agents by using the concepts underlying the ABC methodology, with the purpose of providing an accurate and relevant economic information for conduction of clinical trials. The results of the study showed that estimated “per patient” cost accounted in average for 11.379 Euro. This cost was obtained by adding the costs of all the activities (pre-study, treatment, monitoring, follow-up, audit, and administrative activities) to the overhead costs share. Pre-study activities (20,71% vs 24,85 without overhead costs share), trial monitoring (6,15% vs 7,38% without overhead costs share) and audit (11,10% vs 13,32% without overhead costs share) accounted for roughly 38% (about 46% if we don’t consider overhead costs share) of the estimated patient’s care cost, while treatment accounted for 42% (this value grows to approximately 50% without overhead costs). It is important to consider that not all costs related to the activities vary with the number of patients. Pre study site visit, trial monitoring and audit are fixed costs and they must be computed not more than once for the total patient cost calculation: therefore, the higher the number of enrolled patients the lower will be their unit cost.

Radiodiagnostic service had an estimated cost higher than medical oncology units (2.901 vs 2.824 Euro), but most of the cost was due to tariff value of diagnostic tests performed, equal to 75% of the total cost (as shown in Fig 3), while only the remaining 25% was related to test interpretation.

Generally, the cost of test interpretation is already included in the reimbursement tariff value, but the interviews with key personnel revealed that the diagnostic interpretation of the tests performed in clinical trials is more complex compared to those performed in ordinary diagnostic medical imaging practice and it may require a longer time, due to particular condition such as pseudo-progression that should be carefully evaluated. The greater complexity of clinical trials with immunotherapeutic agents could explain their higher costs compared to clinical trials with target based agents (+22%), mostly related to a greater number of cycles and restaging activities.

In addition to the cost data, the model provides additional information, including: the duration and the number of times that a particular activity has been performed, the number of tests and procedures required by the studies, the possibility to verify the composition of labor cost, i.e., for professional profile and/or specific contractual type, the incidence of personnel cost and the weight of exams on the overall cost, the organizational unit that has supported the larger effort.

A few data on the costs of clinical trials are available in literature. Emanuel EJ and collaborators [9] indicated that, excluding the overhead expenses, a clinical trial on average costs slightly more than 6,094 Dollars (ranging from 2,098 to 19,285) “per enrolled patient” for an industry-sponsored trial, including 1,999 Dollars devoted to nonclinical costs. M.D. Anderson Cancer Center showed that the average cost for treating patients enrolled in clinical trials was 13,802 Dollars for ovarian cancer, 15,650 Dollars per patient for lung cancer, 16,775 Dollars for prostate cancer [10]. In contrast, a study of four companies found that the “per patient” costs for an industry-sponsored study ranged from 60,000 to 85,000 Dollars for Phase III studies [11]. In the study of Sertkaya A and collaborators, aggregate data from three proprietary databases on clinical trial costs provided by Medidata Solutions were used [12]. The three databases showed that the top three cost drivers of clinical trial expenditures were clinical procedure costs (15%–22% of total), administrative staff costs (11%–29% of total), and site monitoring costs (9%–14% of total), excluding estimated site overhead costs and costs for sponsors to monitor the study. A comprehensive cost item list was exemplified by Speich B et al. through a retrospective assessment of resource used and costs in two investigator-initiated randomized trials conducted in Switzerland and Tanzania [13]. The resource used was empirically assessed in a standardized manner through semi-structured interviews and a systematically developed cost item list. The assessed cost data indicated that the patient enrollment, treatment, and follow-up phase represented in each case the most expensive part (84.2% and 46.3%, respectively), as in our study. However, both trials were not conducted with oncological drugs and the authors included in the analysis further items such as trial conception, planning and preparation that may not be generalizable to trials funded by pharmaceutical companies and that were not evaluated in our study.

The present study has several potential limitations. First of all, “per patient” cost was estimated based on protocol procedures rather than measured. Secondly, we obtained information regarding the time to perform activity through key-staffs interviews method. Thus, the time could be overestimated or underestimated. Moreover, considering that not all costs are variable, it is to be expected that as the number of enrolled patients increases, their unit cost decrease. Finally, actual costs of the drugs were not calculated in our study, because they were entirely supplied by the Sponsor.

In conclusion, the evaluation of the cost of a clinical trial is a complex activity. In the present study we assessed the estimated “per patient” total cost for an Oncologic Italian Cancer Center participating in a multicenter clinical trial with new anticancer biological agents through the ABC methodology that is a model for separating clinical trial costs from those related to other clinical activities, looking for an impartial system to ensure a fair remuneration for services required to cost centers (such as those provided by radiology, clinical laboratory, cardiology or pathology). Therefore, this study could allow a better understanding of the nature of the costs produced by clinical trials, providing data and information previously unknown and not available by using traditional cost-center-based accounting systems, that help to improve decision making processes. However, this model can be used to better understand the expenses produced by clinical trials also in non Oncologic settings, to estimate a reliable budget before starting a trial and to support the Hospital in solving issues related to the daily clinical activities and to the costs of the provided services.

## Supporting information

S1 Table Primary activities. (DOCX)

S2 Table Expense-Activity-Link Matrix (EAL-matrix). (DOCX)

S3 Table EAL matrix. (DOCX)

S4 Table Clinical trials of ovarian and urothelial medical oncology. (DOCX)

S5 Table Clinical trials of thoracic medical oncology. (DOCX)

S6 Table Clinical trials of melanoma medical oncology. (DOCX)

S7 Table Costs of clinical trials with Parp Inhibitor (7a), anti PD-L1 Inhibitor (7b) and Parp Inhibitor (7c).
(XLSX)

S8 Table Costs of clinical trials with Anti-PD-1 Inhibitor (8a), anti EGFR Inhibitor (8b) and anti EGFR Inhibitor (8c).
(XLSX)

S9 Table Costs of clinical trials with Anti-PD-1 Inhibitor (9a), anti PD-1 Inhibitor (9b) and BRAF Inhibitor (9c).
(XLSX)

S10 Table Time spent in clinical trials with Parp Inhibitor (10a), anti PD-L1 Inhibitor (10b) and Parp Inhibitor (10c).
(XLSX)

S11 Table Time spent in clinical trials with Anti-PD-1 Inhibitor (11a), anti EGFR Inhibitor (11b) and anti EGFR Inhibitor (11c).
(XLSX)

S12 Table Time spent in clinical trials Anti-PD-1 Inhibitor (12a), anti PD-1 Inhibitor (12b) and BRAF Inhibitor (12c).
(XLSX)

S13 Table Hour rate min and max. (XLSX)

S14 Table Tests cost. (XLSX)

S15 Table Example of collected data of a clinical trial used to estimate the”per patient” total cost. (XLSX)

S1 Text Methodology of the research. (DOCX)
