# Supplementary material for: Costs of clinical trials with anticancer biological agents in an Oncologic Italian Cancer Center using the activity-based costing methodology
Source: PLoS One. 2019 Jan 8;14(1):e0210330. doi: 10.1371/journal.pone.0210330 (PMC6324822; doi:10.1371/journal.pone.0210330)
Supplement: S1 Table — (DOCX) [file pone.0210330.s001.docx]

**S1 Table. Primary activities**

| **PRE STUDY** |
| --- |
| REVIEW THE ADEQUACY OF THE SITE |
| SITE INITIATION VISIT-CARDIOLOGY VISIT |
| SITE INITIATION VISIT-LABORATORY VISIT |
| SITE INITIATION VISIT-PATHOLOGY VISIT |
| SITE INITIATION VISIT-PHARMACY VISIT |
| SITE INITIATION VISIT-PROPOSAL AND SIGN OF STUDY PROTOCOL |
| SITE INITIATION VISIT-RADIODIAGNOSTIC 1 SERVICE VISIT |
| SITE INITIATION VISIT-RADIODIAGNOSTIC 2 SERVICE VISIT |
| **SCREENING** |
| BLOOD SAMPLE AND LABORATORY TESTS |
| CARDIOLOGIC TEST INTERPRETATION AND DOCUMENTAZION IN HEALTH RECORD |
| CARDIOLOGICAL VISIT AND ELECTROCARDIOGRAM |
| COMMUNICATION TO THE PATIENT OF THE TESTS DATE |
| DATA ENTRY IN THE SPONSOR SITE |
| EXTERNAL TEST INTERPRETATION |
| ISTOLOGICAL EXAM |
| LABORATORY TEST INTERPRETATION AND DOCUMENTATION IN HEALTH RECORD |
| ONCOLOGICAL VISIT AND DOCUMENTATION IN HEALTH RECORD |
| OTHER EXAMS (EVENTUALLY PROVIDED BY THE PROTOCOL) |
| OTHERS TEST INTERPRETATION AND DOCUMENTATION IN HEALTH RECORD |
| PATIENT ASSESSMENT |
| PLANNING OF DIAGNOSTIC ACTIVITY (TESTS AND MEDICAL EXAMINATIONS) |
| RADIOLOGIC TEST INTERPRETATION AND DOCUMENTAZION IN HEALTH RECORD |
| RADIOLOGICAL EXAMINATION |
| SHIPPING ISTOLOGICAL SAMPLES TO THE SPONSOR |
| VITAL SIGNS EVALUATION AND DOCUMENTATION IN HEALTH RECORD |
| VITAL SIGNS MEASUREMENT AND DOCUMENTATION IN HEALTH RECORD |
| **AUDIT** |
| IF PROVIDED BY THE STUDY |
| **ENROLLMENT** |
| IVRS CALL |
| MEDICAL RECORD SET-UP |
| PATIENT IDENTIFICATION |
| PATIENT TELEPHONE CALL |
| SIGNATURE INFORMED CONSENT |
| **RANDOMIZATION** |
| IVRS CALL |
| **CYCLE 1** |
| BLOOD SAMPLE AND LABORATORY TESTS |
| CLINICAL TRIAL PATIENT FEEDBACK QUESTIONNAIRE |
| DATA ENTRY IN THE SPONSOR SITE |
| DRUG ADMINISTRATION |
| DRUG RECEPTION AND IVRS CALL |
| EXPERIMENTAL DRUG PREPARATION |
| EXTERNAL TEST INTERPRETATION |
| LABORATORY TEST INTERPRETATION AND DOCUMENTATION IN HEALTH RECORD |
| ONCOLOGICAL VISIT AND DOCUMENTATION IN HEALTH RECORD |
| ONCOLOGICAL VISIT, DISCHARGE AND DOCUMENTATION IN HEALTH RECORD |
| RECEIVING, MANAGEMENT AND SENDING EXPERIMENTAL DRUG |
| VITAL SIGNS EVALUATION AND DOCUMENTATION IN HEALTH RECORD |
| VITAL SIGNS MEASUREMENT AND DOCUMENTATION IN HEALTH RECORD |
| **CYCLE 2** |
| BLOOD SAMPLE AND LABORATORY TESTS |
| CLINICAL TRIAL PATIENT FEEDBACK QUESTIONNAIRE |
| DATA ENTRY IN THE SPONSOR SITE |
| DRUG ADMINISTRATION |
| DRUG RECEPTION AND IVRS CALL |
| EXPERIMENTAL DRUG PREPARATION |
| LABORATORY TEST INTERPRETATION AND DOCUMENTATION IN HEALTH RECORD |
| ONCOLOGICAL VISIT AND DOCUMENTATION IN HEALTH RECORD |
| ONCOLOGICAL VISIT, DISCHARGE AND DOCUMENTATION IN HEALTH RECORD |
| RECEIVING, MANAGEMENT AND SENDING EXPERIMENTAL DRUG |
| THERAPY DAY ORGANIZATION AND PATIENT COMMUNICATION |
| VITAL SIGNS EVALUATION AND DOCUMENTATION IN HEALTH RECORD |
| VITAL SIGNS MEASUREMENT AND DOCUMENTATION IN HEALTH RECORD |
| **CYCLE 3** |
| BLOOD SAMPLE AND LABORATORY TESTS |
| CLINICAL TRIAL PATIENT FEEDBACK QUESTIONNAIRE |
| DATA ENTRY IN THE SPONSOR SITE |
| DRUG ADMINISTRATION |
| DRUG RECEPTION AND IVRS CALL |
| EXPERIMENTAL DRUG PREPARATION |
| LABORATORY TEST INTERPRETATION AND DOCUMENTATION IN HEALTH RECORD |
| ONCOLOGICAL VISIT AND DOCUMENTATION IN HEALTH RECORD |
| ONCOLOGICAL VISIT, DISCHARGE AND DOCUMENTATION IN HEALTH RECORD |
| RECEIVING, MANAGEMENT AND SENDING EXPERIMENTAL DRUG |
| THERAPY DAY ORGANIZATION AND PATIENT COMMUNICATION |
| VITAL SIGNS EVALUATION AND DOCUMENTATION IN HEALTH RECORD |
| VITAL SIGNS MEASUREMENT AND DOCUMENTATION IN HEALTH RECORD |
| **1st RESTAGING** |
| DATA ENTRY IN THE SPONSOR SITE |
| PLANNING OF DIAGNOSTIC ACTIVITY (TESTS AND MEDICAL EXAMINATIONS) |
| RADIOLOGIC TEST INTERPRETATION AND DOCUMENTAZION IN HEALTH RECORD |
| RADIOLOGICAL EXAMINATION |
| (vuoto) |
| **CYCLE 4** |
| BLOOD SAMPLE AND LABORATORY TESTS |
| CLINICAL TRIAL PATIENT FEEDBACK QUESTIONNAIRE |
| DATA ENTRY IN THE SPONSOR SITE |
| DRUG ADMINISTRATION |
| DRUG RECEPTION AND IVRS CALL |
| EXPERIMENTAL DRUG PREPARATION |
| LABORATORY TEST INTERPRETATION AND DOCUMENTATION IN HEALTH RECORD |
| ONCOLOGICAL VISIT AND DOCUMENTATION IN HEALTH RECORD |
| ONCOLOGICAL VISIT, DISCHARGE AND DOCUMENTATION IN HEALTH RECORD |
| RECEIVING, MANAGEMENT AND SENDING EXPERIMENTAL DRUG |
| THERAPY DAY ORGANIZATION AND PATIENT COMMUNICATION |
| VITAL SIGNS EVALUATION AND DOCUMENTATION IN HEALTH RECORD |
| VITAL SIGNS MEASUREMENT AND DOCUMENTATION IN HEALTH RECORD |
| **CYCLE 5** |
| BLOOD SAMPLE AND LABORATORY TESTS |
| CLINICAL TRIAL PATIENT FEEDBACK QUESTIONNAIRE |
| DATA ENTRY IN THE SPONSOR SITE |
| DRUG ADMINISTRATION |
| DRUG RECEPTION AND IVRS CALL |
| EXPERIMENTAL DRUG PREPARATION |
| LABORATORY TEST INTERPRETATION AND DOCUMENTATION IN HEALTH RECORD |
| ONCOLOGICAL VISIT AND DOCUMENTATION IN HEALTH RECORD |
| ONCOLOGICAL VISIT, DISCHARGE AND DOCUMENTATION IN HEALTH RECORD |
| RECEIVING, MANAGEMENT AND SENDING EXPERIMENTAL DRUG |
| THERAPY DAY ORGANIZATION AND PATIENT COMMUNICATION |
| VITAL SIGNS EVALUATION AND DOCUMENTATION IN HEALTH RECORD |
| VITAL SIGNS MEASUREMENT AND DOCUMENTATION IN HEALTH RECORD |
| **CYCLE 6** |
| BLOOD SAMPLE AND LABORATORY TESTS |
| CLINICAL TRIAL PATIENT FEEDBACK QUESTIONNAIRE |
| DATA ENTRY IN THE SPONSOR SITE |
| DRUG ADMINISTRATION |
| DRUG RECEPTION AND IVRS CALL |
| EXPERIMENTAL DRUG PREPARATION |
| LABORATORY TEST INTERPRETATION AND DOCUMENTATION IN HEALTH RECORD |
| ONCOLOGICAL VISIT AND DOCUMENTATION IN HEALTH RECORD |
| ONCOLOGICAL VISIT, DISCHARGE AND DOCUMENTATION IN HEALTH RECORD |
| RECEIVING, MANAGEMENT AND SENDING EXPERIMENTAL DRUG |
| THERAPY DAY ORGANIZATION AND PATIENT COMMUNICATION |
| VITAL SIGNS EVALUATION AND DOCUMENTATION IN HEALTH RECORD |
| VITAL SIGNS MEASUREMENT AND DOCUMENTATION IN HEALTH RECORD |
| **2st RESTAGING** |
| DATA ENTRY IN THE SPONSOR SITE |
| PLANNING OF DIAGNOSTIC ACTIVITY (TESTS AND MEDICAL EXAMINATIONS) |
| RADIOLOGIC TEST INTERPRETATION AND DOCUMENTAZION IN HEALTH RECORD |
| RADIOLOGICAL EXAMINATION |
| (vuoto) |
| **CYCLE 7** |
| BLOOD SAMPLE AND LABORATORY TESTS |
| CLINICAL TRIAL PATIENT FEEDBACK QUESTIONNAIRE |
| DATA ENTRY IN THE SPONSOR SITE |
| DRUG ADMINISTRATION |
| DRUG RECEPTION AND IVRS CALL |
| EXPERIMENTAL DRUG PREPARATION |
| LABORATORY TEST INTERPRETATION AND DOCUMENTATION IN HEALTH RECORD |
| ONCOLOGICAL VISIT AND DOCUMENTATION IN HEALTH RECORD |
| ONCOLOGICAL VISIT, DISCHARGE AND DOCUMENTATION IN HEALTH RECORD |
| RECEIVING, MANAGEMENT AND SENDING EXPERIMENTAL DRUG |
| THERAPY DAY ORGANIZATION AND PATIENT COMMUNICATION |
| VITAL SIGNS EVALUATION AND DOCUMENTATION IN HEALTH RECORD |
| VITAL SIGNS MEASUREMENT AND DOCUMENTATION IN HEALTH RECORD |
| **CYCLE 8** |
| BLOOD SAMPLE AND LABORATORY TESTS |
| CLINICAL TRIAL PATIENT FEEDBACK QUESTIONNAIRE |
| DATA ENTRY IN THE SPONSOR SITE |
| DRUG ADMINISTRATION |
| DRUG RECEPTION AND IVRS CALL |
| EXPERIMENTAL DRUG PREPARATION |
| LABORATORY TEST INTERPRETATION AND DOCUMENTATION IN HEALTH RECORD |
| ONCOLOGICAL VISIT AND DOCUMENTATION IN HEALTH RECORD |
| ONCOLOGICAL VISIT, DISCHARGE AND DOCUMENTATION IN HEALTH RECORD |
| RECEIVING, MANAGEMENT AND SENDING EXPERIMENTAL DRUG |
| THERAPY DAY ORGANIZATION AND PATIENT COMMUNICATION |
| VITAL SIGNS EVALUATION AND DOCUMENTATION IN HEALTH RECORD |
| VITAL SIGNS MEASUREMENT AND DOCUMENTATION IN HEALTH RECORD |
| **CYCLE 9** |
| BLOOD SAMPLE AND LABORATORY TESTS |
| CLINICAL TRIAL PATIENT FEEDBACK QUESTIONNAIRE |
| DATA ENTRY IN THE SPONSOR SITE |
| DRUG ADMINISTRATION |
| DRUG RECEPTION AND IVRS CALL |
| EXPERIMENTAL DRUG PREPARATION |
| LABORATORY TEST INTERPRETATION AND DOCUMENTATION IN HEALTH RECORD |
| ONCOLOGICAL VISIT AND DOCUMENTATION IN HEALTH RECORD |
| ONCOLOGICAL VISIT, DISCHARGE AND DOCUMENTATION IN HEALTH RECORD |
| RECEIVING, MANAGEMENT AND SENDING EXPERIMENTAL DRUG |
| THERAPY DAY ORGANIZATION AND PATIENT COMMUNICATION |
| VITAL SIGNS EVALUATION AND DOCUMENTATION IN HEALTH RECORD |
| VITAL SIGNS MEASUREMENT AND DOCUMENTATION IN HEALTH RECORD |
| **3st RESTAGING** |
| DATA ENTRY IN THE SPONSOR SITE |
| PLANNING OF DIAGNOSTIC ACTIVITY (TESTS AND MEDICAL EXAMINATIONS) |
| RADIOLOGIC TEST INTERPRETATION AND DOCUMENTAZION IN HEALTH RECORD |
| RADIOLOGICAL EXAMINATION |
| (vuoto) |
| **CYCLE 10** |
| BLOOD SAMPLE AND LABORATORY TESTS |
| CLINICAL TRIAL PATIENT FEEDBACK QUESTIONNAIRE |
| DATA ENTRY IN THE SPONSOR SITE |
| DRUG ADMINISTRATION |
| DRUG RECEPTION AND IVRS CALL |
| EXPERIMENTAL DRUG PREPARATION |
| LABORATORY TEST INTERPRETATION AND DOCUMENTATION IN HEALTH RECORD |
| ONCOLOGICAL VISIT AND DOCUMENTATION IN HEALTH RECORD |
| ONCOLOGICAL VISIT, DISCHARGE AND DOCUMENTATION IN HEALTH RECORD |
| RECEIVING, MANAGEMENT AND SENDING EXPERIMENTAL DRUG |
| THERAPY DAY ORGANIZATION AND PATIENT COMMUNICATION |
| VITAL SIGNS EVALUATION AND DOCUMENTATION IN HEALTH RECORD |
| VITAL SIGNS MEASUREMENT AND DOCUMENTATION IN HEALTH RECORD |
| **CYCLE 11** |
| BLOOD SAMPLE AND LABORATORY TESTS |
| CLINICAL TRIAL PATIENT FEEDBACK QUESTIONNAIRE |
| DATA ENTRY IN THE SPONSOR SITE |
| DRUG ADMINISTRATION |
| DRUG RECEPTION AND IVRS CALL |
| EXPERIMENTAL DRUG PREPARATION |
| LABORATORY TEST INTERPRETATION AND DOCUMENTATION IN HEALTH RECORD |
| ONCOLOGICAL VISIT AND DOCUMENTATION IN HEALTH RECORD |
| ONCOLOGICAL VISIT, DISCHARGE AND DOCUMENTATION IN HEALTH RECORD |
| RECEIVING, MANAGEMENT AND SENDING EXPERIMENTAL DRUG |
| THERAPY DAY ORGANIZATION AND PATIENT COMMUNICATION |
| VITAL SIGNS EVALUATION AND DOCUMENTATION IN HEALTH RECORD |
| VITAL SIGNS MEASUREMENT AND DOCUMENTATION IN HEALTH RECORD |
| **CYCLE 12** |
| BLOOD SAMPLE AND LABORATORY TESTS |
| CLINICAL TRIAL PATIENT FEEDBACK QUESTIONNAIRE |
| DATA ENTRY IN THE SPONSOR SITE |
| DRUG ADMINISTRATION |
| DRUG RECEPTION AND IVRS CALL |
| EXPERIMENTAL DRUG PREPARATION |
| LABORATORY TEST INTERPRETATION AND DOCUMENTATION IN HEALTH RECORD |
| ONCOLOGICAL VISIT AND DOCUMENTATION IN HEALTH RECORD |
| ONCOLOGICAL VISIT, DISCHARGE AND DOCUMENTATION IN HEALTH RECORD |
| RECEIVING, MANAGEMENT AND SENDING EXPERIMENTAL DRUG |
| THERAPY DAY ORGANIZATION AND PATIENT COMMUNICATION |
| VITAL SIGNS EVALUATION AND DOCUMENTATION IN HEALTH RECORD |
| VITAL SIGNS MEASUREMENT AND DOCUMENTATION IN HEALTH RECORD |
| **4st RESTAGING** |
| DATA ENTRY IN THE SPONSOR SITE |
| PLANNING OF DIAGNOSTIC ACTIVITY (TESTS AND MEDICAL EXAMINATIONS) |
| RADIOLOGIC TEST INTERPRETATION AND DOCUMENTAZION IN HEALTH RECORD |
| RADIOLOGICAL EXAMINATION |
| (vuoto) |
| **1st TRIAL MONITORING** |
| OVERSEEING THE PROGRESS OF THE CLINICAL TRIAL |
| **EXIT CANCER VISIT** |
| BLOOD SAMPLE AND LABORATORY TESTS |
| CLINICAL TRIAL PATIENT FEEDBACK QUESTIONNAIRE |
| DATA ENTRY IN THE SPONSOR SITE |
| IVRS CALL FOR EXIT PATIENT |
| LABORATORY TEST INTERPRETATION AND DOCUMENTATION IN HEALTH RECORD |
| ONCOLOGICAL VISIT AND DOCUMENTATION IN HEALTH RECORD |
| ONCOLOGICAL VISIT, DISCHARGE AND DOCUMENTATION IN HEALTH RECORD |
| PLANNING OF DIAGNOSTIC ACTIVITY (TESTS AND MEDICAL EXAMINATIONS) |
| VITAL SIGNS EVALUATION AND DOCUMENTATION IN HEALTH RECORD |
| VITAL SIGNS MEASUREMENT AND DOCUMENTATION IN HEALTH RECORD |
| **35-DAY FOLLOW-UP** |
| BLOOD SAMPLE AND LABORATORY TESTS |
| CLINICAL TRIAL PATIENT FEEDBACK QUESTIONNAIRE |
| DATA ENTRY IN THE SPONSOR SITE |
| LABORATORY TEST INTERPRETATION AND DOCUMENTATION IN HEALTH RECORD |
| ONCOLOGICAL VISIT AND DOCUMENTATION IN HEALTH RECORD |
| ONCOLOGICAL VISIT, DISCHARGE AND DOCUMENTATION IN HEALTH RECORD |
| PLANNING OF DIAGNOSTIC ACTIVITY (TESTS AND MEDICAL EXAMINATIONS) |
| VITAL SIGNS EVALUATION AND DOCUMENTATION IN HEALTH RECORD |
| VITAL SIGNS MEASUREMENT AND DOCUMENTATION IN HEALTH RECORD |
| **90-DAY FOLLOW-UP** |
| BLOOD SAMPLE AND LABORATORY TESTS |
| CLINICAL TRIAL PATIENT FEEDBACK QUESTIONNAIRE |
| DATA ENTRY IN THE SPONSOR SITE |
| LABORATORY TEST INTERPRETATION AND DOCUMENTATION IN HEALTH RECORD |
| ONCOLOGICAL VISIT AND DOCUMENTATION IN HEALTH RECORD |
| ONCOLOGICAL VISIT, DISCHARGE AND DOCUMENTATION IN HEALTH RECORD |
| PLANNING OF DIAGNOSTIC ACTIVITY (TESTS AND MEDICAL EXAMINATIONS) |
| VITAL SIGNS EVALUATION AND DOCUMENTATION IN HEALTH RECORD |
| VITAL SIGNS MEASUREMENT AND DOCUMENTATION IN HEALTH RECORD |
| **1st SURVIVAL FOLLOW-UP** |
| CLINICAL TRIAL PATIENT FEEDBACK QUESTIONNAIRE |
| DATA ENTRY IN THE SPONSOR SITE |
| ONCOLOGICAL VISIT AND DOCUMENTATION IN HEALTH RECORD |
| ONCOLOGICAL VISIT, DISCHARGE AND DOCUMENTATION IN HEALTH RECORD |
| PLANNING OF DIAGNOSTIC ACTIVITY (TESTS AND MEDICAL EXAMINATIONS) |
| VITAL SIGNS EVALUATION AND DOCUMENTATION IN HEALTH RECORD |
| VITAL SIGNS MEASUREMENT AND DOCUMENTATION IN HEALTH RECORD |
| **2st SURVIVAL FOLLOW-UP** |
| CLINICAL TRIAL PATIENT FEEDBACK QUESTIONNAIRE |
| DATA ENTRY IN THE SPONSOR SITE |
| ONCOLOGICAL VISIT AND DOCUMENTATION IN HEALTH RECORD |
| ONCOLOGICAL VISIT, DISCHARGE AND DOCUMENTATION IN HEALTH RECORD |
| PLANNING OF DIAGNOSTIC ACTIVITY (TESTS AND MEDICAL EXAMINATIONS) |
| VITAL SIGNS EVALUATION AND DOCUMENTATION IN HEALTH RECORD |
| VITAL SIGNS MEASUREMENT AND DOCUMENTATION IN HEALTH RECORD |
| **ADMINISTRATIVE ACTIVITY** |
| ADMINISTRATIVE ACTIVITY |
| INVOICING FOR TRIAL PAYMENTS |
| **Total** |
|  |
