# Supplementary material for: Costs of clinical trials with anticancer biological agents in an Oncologic Italian Cancer Center using the activity-based costing methodology
Source: PLoS One. 2019 Jan 8;14(1):e0210330. doi: 10.1371/journal.pone.0210330 (PMC6324822; doi:10.1371/journal.pone.0210330)
Supplement: S3 Table — (DOCX) [file pone.0210330.s003.docx]

**S3 Table - Bridging EAL matrix**

| **ACTIVITY POOL** | **MEDICAL ONCOLOGIES** | **RADIODIAGNOSTIC** | **NUCLEAR MEDICINE** | **PHARMACY** | **CLINICAL LABORATORY** | **PATHOLOGICAL ANATOMY** | **CARDIOLOGY** | **MOLECULAR BIOLOGY** | **ACTIVITY COST POOL** |
| --- | --- | --- | --- | --- | --- | --- | --- | --- | --- |
| PRE-STUDY ACTIVITIES |  |  |  |  |  |  |  |  |  |
| TREATMENT |  |  |  |  |  |  |  |  |  |
| TRIAL MONITORING |  |  |  |  |  |  |  |  |  |
| FOLLOW-UP |  |  |  |  |  |  |  |  |  |
| AUDIT |  |  |  |  |  |  |  |  |  |
| ADMINISTRATIVE ACTIVITY |  |  |  |  |  |  |  |  |  |
| **ESTIMATED TOTAL COST** |  |  |  |  |  |  |  |  |  |
| OVERHEAD COST (20%) | | | | | | | | |  |
| **ESTIMATED TRIAL TOTAL COST** | | | | | | | | |  |
