# Supplementary material for: Costs of clinical trials with anticancer biological agents in an Oncologic Italian Cancer Center using the activity-based costing methodology
Source: PLoS One. 2019 Jan 8;14(1):e0210330. doi: 10.1371/journal.pone.0210330 (PMC6324822; doi:10.1371/journal.pone.0210330)
Supplement: S4 Table — (DOCX) [file pone.0210330.s004.docx]

S4 Table. Clinical trials of ovarian and urothelial Medical Oncology
