# Supplementary material for: Costs of clinical trials with anticancer biological agents in an Oncologic Italian Cancer Center using the activity-based costing methodology
Source: PLoS One. 2019 Jan 8;14(1):e0210330. doi: 10.1371/journal.pone.0210330 (PMC6324822; doi:10.1371/journal.pone.0210330)
Supplement: S1 Text — (DOCX) [file pone.0210330.s016.docx]

**Methodology of the research**

We used a semi-structured interview, following the below steps:

1. We first asked the principal investigators (PI) who participated in this project to identify randomized clinical trials conducted at their Units with immunotherapeutic and target based agents and the name of the equipe of the studies. The investigators identified the following trials: ARIEL 3, MK3475-045, SOLO1, CheckMate-026, BR26, U31287-A-U301, CA209-172, MK3475-002 and MO25515.
2. Then, we asked the PI and the study coordinators of the studies to describe all the single phases of the clinical trials, leading to define a “patient clinical pathway” (as reported in figure 1).
3. Thereafter, we minutely reconstructed all the phases of each clinical trial, building a table of main activities (shown below in original form and in English, as requested):

| **ACTIVITY POOL E RELATIVE ATTIVITA' PRINCIPALI** |
| --- |
| **Etichette di riga** |
| **PRE STUDY** |
| SIV: PRESENTAZIONE STUDIO E FIRMA DOCUMENTAZIONE |
| SIV: VISITA CARDIOLOGIA |
| SIV: VISITA FARMACIA |
| SIV: VISITA MEDICINA DI LABORATORIO |
| SIV: VISITA RADIODIAGNOSTICA DAY HOSPITAL |
| SIV: VISITA RADIODIAGNOSTICA DEGENZE |
| VALUTAZIONE IDONIETA' STRUTTURA |
| SIV: VISITA ANATOMIA PATOLOGICA |
| **ARRUOLAMENTO PAZIENTE** |
| APERTURA DIARIO CLINICO |
| CHIAMATA IN IVRS (DATABASE) |
| CONTATTO DELLA STRUTTURA DA PARTE DEL PAZIENTE |
| FIRMA DEL CONSENSO INFORMATO |
| INDIVIDUAZIONE DEL PAZIENTE |
| **SCREENING** |
| ALTRI ESAMI (EVENTUALI PREVISTI DAL PROTOCOLLO) |
| COMUNICAZIONE AL PAZIENTE DELLE DATE DI ESAME |
| ESAMI RADIOLOGICI |
| INSERIMENTO DATI NEL DATABASE |
| PRELIEVO ED ESAMI DI LABORATORIO |
| PROGRAMMAZIONE ATTIVITÀ DIAGNOSTICA (ESAMI E VISITE SPECIALISTICHE) |
| RACCOLTA E ANNOTAZIONE PARAMETRI VITALI |
| REFERTAZIONE/RI-REFERTAZIONE DI ESAMI ESTERNI |
| VALUTAZIONE DELLO SCREENING (CONDIZIONI GENERALI DEL PAZIENTE) |
| VALUTAZIONE ESAMI DI LABORATORIO E ANNOTAZIONE NEL DIARIO CLINICO |
| VALUTAZIONE ESITO ALTRI ESAMI E ANNOTAZIONE NEL DIARIO CLINICO |
| VALUTAZIONE ESITO INDAGINE RADIOLOGICA E ANNOTAZIONE NEL DIARIO CLINICO |
| VALUTAZIONE ESITO VISITA CARDIOLOGICA E ANNOTAZIONE NEL DIARIO CLINICO |
| VALUTAZIONE PARAMETRI VITALI E ANNOTAZIONE NEL DIARIO CLINICO |
| VISITA CARDIOLOGICA E ECG |
| VISITA ONCOLOGICA E ANNOTAZIONE NEL DIARIO CLINICO |
| ESAME ISTOLOGICO |
| SPEDIZIONE CAMPIONI ISTOLOGICI ALLO SPONSOR |
| **RANDOMIZZAZIONE** |
| CHIAMATA IN IVRS |
| **VISITA FINALE PER USCITA** |
| CHIAMATA IN IVRS PER USCITA PAZIENTE |
| INSERIMENTO DATI NEL DATABASE |
| PRELIEVO ED ESAMI DI LABORATORIO |
| PROGRAMMAZIONE ATTIVITÀ DIAGNOSTICA (ESAMI E VISITE SPECIALISTICHE) |
| RACCOLTA E ANNOTAZIONE PARAMETRI VITALI |
| SOMMINISTRAZIONE QUESTIONARIO |
| VALUTAZIONE ESAMI DI LABORATORIO E ANNOTAZIONE NEL DIARIO CLINICO |
| VALUTAZIONE PARAMETRI VITALI E ANNOTAZIONE NEL DIARIO CLINICO |
| VISITA FINALE, DIMISSIONE E ANNOTAZIONE NEL DIARIO CLINICO |
| VISITA ONCOLOGICA E ANNOTAZIONE NEL DIARIO CLINICO |
| **ATTIVITA' AMMINISTRATIVA** |
| ATTIVITA' DELLA SC AMMINISTRATIVA DELLA RICERCA |
| RENDICONTAZIONE FATTURE STUDIO CLINICO |
| **AUDIT** |
| SE PREVISTO |
| **CICLO 1** |
| INSERIMENTO DATI NEL DATABASE |
| PRELIEVO ED ESAMI DI LABORATORIO |
| PREPARAZIONE/ALLESTIMENTO UMACA FARMACO SPERIMENTALE |
| PROGRAMMAZIONE E COMUNICAZIONE DATA GIORNATA DI TERAPIA |
| RACCOLTA E ANNOTAZIONE PARAMETRI VITALI |
| RICEZIONE FARMACO SPERIMENTALE E COMUNICAZIONE IN IVRS |
| RICEZIONE/GESTIONE/INVIO FARMACO SPERIMENTALE |
| SOMMINISTAZIONE FARMACO |
| SOMMINISTRAZIONE QUESTIONARIO |
| VALUTAZIONE ESAMI DI LABORATORIO E ANNOTAZIONE NEL DIARIO CLINICO |
| VALUTAZIONE PARAMETRI VITALI E ANNOTAZIONE NEL DIARIO CLINICO |
| VISITA FINALE, DIMISSIONE E ANNOTAZIONE NEL DIARIO CLINICO |
| VISITA ONCOLOGICA E ANNOTAZIONE NEL DIARIO CLINICO |
| **CICLO 2** |
| INSERIMENTO DATI NEL DATABASE |
| PRELIEVO ED ESAMI DI LABORATORIO |
| PREPARAZIONE/ALLESTIMENTO UMACA FARMACO SPERIMENTALE |
| PROGRAMMAZIONE E COMUNICAZIONE DATA GIORNATA DI TERAPIA |
| RACCOLTA E ANNOTAZIONE PARAMETRI VITALI |
| RICEZIONE FARMACO SPERIMENTALE E COMUNICAZIONE IN IVRS |
| RICEZIONE/GESTIONE/INVIO FARMACO SPERIMENTALE |
| SOMMINISTAZIONE FARMACO |
| SOMMINISTRAZIONE QUESTIONARIO |
| VALUTAZIONE ESAMI DI LABORATORIO E ANNOTAZIONE NEL DIARIO CLINICO |
| VALUTAZIONE PARAMETRI VITALI E ANNOTAZIONE NEL DIARIO CLINICO |
| VISITA FINALE, DIMISSIONE E ANNOTAZIONE NEL DIARIO CLINICO |
| VISITA ONCOLOGICA E ANNOTAZIONE NEL DIARIO CLINICO |
| **CICLO 3** |
| INSERIMENTO DATI NEL DATABASE |
| PRELIEVO ED ESAMI DI LABORATORIO |
| PREPARAZIONE/ALLESTIMENTO UMACA FARMACO SPERIMENTALE |
| PROGRAMMAZIONE E COMUNICAZIONE DATA GIORNATA DI TERAPIA |
| RACCOLTA E ANNOTAZIONE PARAMETRI VITALI |
| RICEZIONE FARMACO SPERIMENTALE E COMUNICAZIONE IN IVRS |
| RICEZIONE/GESTIONE/INVIO FARMACO SPERIMENTALE |
| SOMMINISTAZIONE FARMACO |
| SOMMINISTRAZIONE QUESTIONARIO |
| VALUTAZIONE ESAMI DI LABORATORIO E ANNOTAZIONE NEL DIARIO CLINICO |
| VALUTAZIONE PARAMETRI VITALI E ANNOTAZIONE NEL DIARIO CLINICO |
| VISITA FINALE, DIMISSIONE E ANNOTAZIONE NEL DIARIO CLINICO |
| VISITA ONCOLOGICA E ANNOTAZIONE NEL DIARIO CLINICO |
| **FOLLOW-UP 35 GG DALL'ULTIMA DOSE** |
| INSERIMENTO DATI NEL DATABASE |
| PRELIEVO ED ESAMI DI LABORATORIO |
| PROGRAMMAZIONE ATTIVITÀ DIAGNOSTICA (ESAMI E VISITE SPECIALISTICHE) |
| RACCOLTA E ANNOTAZIONE PARAMETRI VITALI |
| SOMMINISTRAZIONE QUESTIONARIO |
| VALUTAZIONE ESAMI DI LABORATORIO E ANNOTAZIONE NEL DIARIO CLINICO |
| VALUTAZIONE PARAMETRI VITALI E ANNOTAZIONE NEL DIARIO CLINICO |
| VISITA FINALE, DIMISSIONE E ANNOTAZIONE NEL DIARIO CLINICO |
| VISITA ONCOLOGICA E ANNOTAZIONE NEL DIARIO CLINICO |
| **FOLLOW-UP A 3 MESI DALL'ULTIMA DOSE** |
| INSERIMENTO DATI NEL DATABASE |
| PRELIEVO ED ESAMI DI LABORATORIO |
| PROGRAMMAZIONE ATTIVITÀ DIAGNOSTICA (ESAMI E VISITE SPECIALISTICHE) |
| RACCOLTA E ANNOTAZIONE PARAMETRI VITALI |
| SOMMINISTRAZIONE QUESTIONARIO |
| VALUTAZIONE ESAMI DI LABORATORIO E ANNOTAZIONE NEL DIARIO CLINICO |
| VALUTAZIONE PARAMETRI VITALI E ANNOTAZIONE NEL DIARIO CLINICO |
| VISITA FINALE, DIMISSIONE E ANNOTAZIONE NEL DIARIO CLINICO |
| VISITA ONCOLOGICA E ANNOTAZIONE NEL DIARIO CLINICO |
| **(vuoto)** |
| (vuoto) |
| **1° RIVALUTAZIONE** |
| ESAMI RADIOLOGICI |
| INSERIMENTO DATI NEL DATABASE |
| PROGRAMMAZIONE ATTIVITÀ DIAGNOSTICA (ESAMI E VISITE SPECIALISTICHE) |
| VALUTAZIONE ESITO INDAGINE RADIOLOGICA E ANNOTAZIONE NEL DIARIO CLINICO |
| (vuoto) |
| TRASMISSIONE DATI SU DATABASE SPONSOR |
| **CICLO 4** |
| INSERIMENTO DATI NEL DATABASE |
| PRELIEVO ED ESAMI DI LABORATORIO |
| PREPARAZIONE/ALLESTIMENTO UMACA FARMACO SPERIMENTALE |
| PROGRAMMAZIONE E COMUNICAZIONE DATA GIORNATA DI TERAPIA |
| RACCOLTA E ANNOTAZIONE PARAMETRI VITALI |
| RICEZIONE FARMACO SPERIMENTALE E COMUNICAZIONE IN IVRS |
| RICEZIONE/GESTIONE/INVIO FARMACO SPERIMENTALE |
| SOMMINISTAZIONE FARMACO |
| SOMMINISTRAZIONE QUESTIONARIO |
| VALUTAZIONE ESAMI DI LABORATORIO E ANNOTAZIONE NEL DIARIO CLINICO |
| VALUTAZIONE PARAMETRI VITALI E ANNOTAZIONE NEL DIARIO CLINICO |
| VISITA FINALE, DIMISSIONE E ANNOTAZIONE NEL DIARIO CLINICO |
| VISITA ONCOLOGICA E ANNOTAZIONE NEL DIARIO CLINICO |
| **CICLO 5** |
| INSERIMENTO DATI NEL DATABASE |
| PRELIEVO ED ESAMI DI LABORATORIO |
| PREPARAZIONE/ALLESTIMENTO UMACA FARMACO SPERIMENTALE |
| PROGRAMMAZIONE E COMUNICAZIONE DATA GIORNATA DI TERAPIA |
| RACCOLTA E ANNOTAZIONE PARAMETRI VITALI |
| RICEZIONE FARMACO SPERIMENTALE E COMUNICAZIONE IN IVRS |
| RICEZIONE/GESTIONE/INVIO FARMACO SPERIMENTALE |
| SOMMINISTAZIONE FARMACO |
| SOMMINISTRAZIONE QUESTIONARIO |
| VALUTAZIONE ESAMI DI LABORATORIO E ANNOTAZIONE NEL DIARIO CLINICO |
| VALUTAZIONE PARAMETRI VITALI E ANNOTAZIONE NEL DIARIO CLINICO |
| VISITA FINALE, DIMISSIONE E ANNOTAZIONE NEL DIARIO CLINICO |
| VISITA ONCOLOGICA E ANNOTAZIONE NEL DIARIO CLINICO |
| **CICLO 6** |
| INSERIMENTO DATI NEL DATABASE |
| PRELIEVO ED ESAMI DI LABORATORIO |
| PREPARAZIONE/ALLESTIMENTO UMACA FARMACO SPERIMENTALE |
| PROGRAMMAZIONE E COMUNICAZIONE DATA GIORNATA DI TERAPIA |
| RACCOLTA E ANNOTAZIONE PARAMETRI VITALI |
| RICEZIONE FARMACO SPERIMENTALE E COMUNICAZIONE IN IVRS |
| RICEZIONE/GESTIONE/INVIO FARMACO SPERIMENTALE |
| SOMMINISTAZIONE FARMACO |
| SOMMINISTRAZIONE QUESTIONARIO |
| VALUTAZIONE ESAMI DI LABORATORIO E ANNOTAZIONE NEL DIARIO CLINICO |
| VALUTAZIONE PARAMETRI VITALI E ANNOTAZIONE NEL DIARIO CLINICO |
| VISITA FINALE, DIMISSIONE E ANNOTAZIONE NEL DIARIO CLINICO |
| VISITA ONCOLOGICA E ANNOTAZIONE NEL DIARIO CLINICO |
| **2° RIVALUTAZIONE** |
| ESAMI RADIOLOGICI |
| INSERIMENTO DATI NEL DATABASE |
| PROGRAMMAZIONE ATTIVITÀ DIAGNOSTICA (ESAMI E VISITE SPECIALISTICHE) |
| VALUTAZIONE ESITO INDAGINE RADIOLOGICA E ANNOTAZIONE NEL DIARIO CLINICO |
| (vuoto) |
| TRASMISSIONE DATI SU DATABASE SPONSOR |
| **CICLO 7** |
| INSERIMENTO DATI NEL DATABASE |
| PRELIEVO ED ESAMI DI LABORATORIO |
| PREPARAZIONE/ALLESTIMENTO UMACA FARMACO SPERIMENTALE |
| PROGRAMMAZIONE E COMUNICAZIONE DATA GIORNATA DI TERAPIA |
| RACCOLTA E ANNOTAZIONE PARAMETRI VITALI |
| RICEZIONE FARMACO SPERIMENTALE E COMUNICAZIONE IN IVRS |
| RICEZIONE/GESTIONE/INVIO FARMACO SPERIMENTALE |
| SOMMINISTAZIONE FARMACO |
| SOMMINISTRAZIONE QUESTIONARIO |
| VALUTAZIONE ESAMI DI LABORATORIO E ANNOTAZIONE NEL DIARIO CLINICO |
| VALUTAZIONE PARAMETRI VITALI E ANNOTAZIONE NEL DIARIO CLINICO |
| VISITA FINALE, DIMISSIONE E ANNOTAZIONE NEL DIARIO CLINICO |
| VISITA ONCOLOGICA E ANNOTAZIONE NEL DIARIO CLINICO |
| **CICLO 8** |
| INSERIMENTO DATI NEL DATABASE |
| PRELIEVO ED ESAMI DI LABORATORIO |
| PREPARAZIONE/ALLESTIMENTO UMACA FARMACO SPERIMENTALE |
| PROGRAMMAZIONE E COMUNICAZIONE DATA GIORNATA DI TERAPIA |
| RACCOLTA E ANNOTAZIONE PARAMETRI VITALI |
| RICEZIONE FARMACO SPERIMENTALE E COMUNICAZIONE IN IVRS |
| RICEZIONE/GESTIONE/INVIO FARMACO SPERIMENTALE |
| SOMMINISTAZIONE FARMACO |
| SOMMINISTRAZIONE QUESTIONARIO |
| VALUTAZIONE ESAMI DI LABORATORIO E ANNOTAZIONE NEL DIARIO CLINICO |
| VALUTAZIONE PARAMETRI VITALI E ANNOTAZIONE NEL DIARIO CLINICO |
| VISITA FINALE, DIMISSIONE E ANNOTAZIONE NEL DIARIO CLINICO |
| VISITA ONCOLOGICA E ANNOTAZIONE NEL DIARIO CLINICO |
| **CICLO 9** |
| INSERIMENTO DATI NEL DATABASE |
| PRELIEVO ED ESAMI DI LABORATORIO |
| PREPARAZIONE/ALLESTIMENTO UMACA FARMACO SPERIMENTALE |
| PROGRAMMAZIONE E COMUNICAZIONE DATA GIORNATA DI TERAPIA |
| RACCOLTA E ANNOTAZIONE PARAMETRI VITALI |
| RICEZIONE FARMACO SPERIMENTALE E COMUNICAZIONE IN IVRS |
| RICEZIONE/GESTIONE/INVIO FARMACO SPERIMENTALE |
| SOMMINISTAZIONE FARMACO |
| SOMMINISTRAZIONE QUESTIONARIO |
| VALUTAZIONE ESAMI DI LABORATORIO E ANNOTAZIONE NEL DIARIO CLINICO |
| VALUTAZIONE PARAMETRI VITALI E ANNOTAZIONE NEL DIARIO CLINICO |
| VISITA FINALE, DIMISSIONE E ANNOTAZIONE NEL DIARIO CLINICO |
| VISITA ONCOLOGICA E ANNOTAZIONE NEL DIARIO CLINICO |
| **3° RIVALUTAZIONE** |
| ESAMI RADIOLOGICI |
| INSERIMENTO DATI NEL DATABASE |
| PROGRAMMAZIONE ATTIVITÀ DIAGNOSTICA (ESAMI E VISITE SPECIALISTICHE) |
| VALUTAZIONE ESITO INDAGINE RADIOLOGICA E ANNOTAZIONE NEL DIARIO CLINICO |
| (vuoto) |
| TRASMISSIONE DATI SU DATABASE SPONSOR |
| **CICLO 10** |
| INSERIMENTO DATI NEL DATABASE |
| PRELIEVO ED ESAMI DI LABORATORIO |
| PREPARAZIONE/ALLESTIMENTO UMACA FARMACO SPERIMENTALE |
| PROGRAMMAZIONE E COMUNICAZIONE DATA GIORNATA DI TERAPIA |
| RACCOLTA E ANNOTAZIONE PARAMETRI VITALI |
| RICEZIONE FARMACO SPERIMENTALE E COMUNICAZIONE IN IVRS |
| RICEZIONE/GESTIONE/INVIO FARMACO SPERIMENTALE |
| SOMMINISTAZIONE FARMACO |
| SOMMINISTRAZIONE QUESTIONARIO |
| VALUTAZIONE ESAMI DI LABORATORIO E ANNOTAZIONE NEL DIARIO CLINICO |
| VALUTAZIONE PARAMETRI VITALI E ANNOTAZIONE NEL DIARIO CLINICO |
| VISITA FINALE, DIMISSIONE E ANNOTAZIONE NEL DIARIO CLINICO |
| VISITA ONCOLOGICA E ANNOTAZIONE NEL DIARIO CLINICO |
| **CICLO 11** |
| INSERIMENTO DATI NEL DATABASE |
| PRELIEVO ED ESAMI DI LABORATORIO |
| PREPARAZIONE/ALLESTIMENTO UMACA FARMACO SPERIMENTALE |
| PROGRAMMAZIONE E COMUNICAZIONE DATA GIORNATA DI TERAPIA |
| RACCOLTA E ANNOTAZIONE PARAMETRI VITALI |
| RICEZIONE FARMACO SPERIMENTALE E COMUNICAZIONE IN IVRS |
| RICEZIONE/GESTIONE/INVIO FARMACO SPERIMENTALE |
| SOMMINISTAZIONE FARMACO |
| SOMMINISTRAZIONE QUESTIONARIO |
| VALUTAZIONE ESAMI DI LABORATORIO E ANNOTAZIONE NEL DIARIO CLINICO |
| VALUTAZIONE PARAMETRI VITALI E ANNOTAZIONE NEL DIARIO CLINICO |
| VISITA FINALE, DIMISSIONE E ANNOTAZIONE NEL DIARIO CLINICO |
| VISITA ONCOLOGICA E ANNOTAZIONE NEL DIARIO CLINICO |
| **CICLO 12** |
| INSERIMENTO DATI NEL DATABASE |
| PRELIEVO ED ESAMI DI LABORATORIO |
| PREPARAZIONE/ALLESTIMENTO UMACA FARMACO SPERIMENTALE |
| PROGRAMMAZIONE E COMUNICAZIONE DATA GIORNATA DI TERAPIA |
| RACCOLTA E ANNOTAZIONE PARAMETRI VITALI |
| RICEZIONE FARMACO SPERIMENTALE E COMUNICAZIONE IN IVRS |
| RICEZIONE/GESTIONE/INVIO FARMACO SPERIMENTALE |
| SOMMINISTAZIONE FARMACO |
| SOMMINISTRAZIONE QUESTIONARIO |
| VALUTAZIONE ESAMI DI LABORATORIO E ANNOTAZIONE NEL DIARIO CLINICO |
| VALUTAZIONE PARAMETRI VITALI E ANNOTAZIONE NEL DIARIO CLINICO |
| VISITA FINALE, DIMISSIONE E ANNOTAZIONE NEL DIARIO CLINICO |
| VISITA ONCOLOGICA E ANNOTAZIONE NEL DIARIO CLINICO |
| **4° RIVALUTAZIONE** |
| ESAMI RADIOLOGICI |
| INSERIMENTO DATI NEL DATABASE |
| PROGRAMMAZIONE ATTIVITÀ DIAGNOSTICA (ESAMI E VISITE SPECIALISTICHE) |
| VALUTAZIONE ESITO INDAGINE RADIOLOGICA E ANNOTAZIONE NEL DIARIO CLINICO |
| (vuoto) |
| TRASMISSIONE DATI SU DATABASE SPONSOR |
| **1° FOLLOW-UP TRIMESTRALI SUCCESSIVI** |
| INSERIMENTO DATI NEL DATABASE |
| PROGRAMMAZIONE ATTIVITÀ DIAGNOSTICA (ESAMI E VISITE SPECIALISTICHE) |
| RACCOLTA E ANNOTAZIONE PARAMETRI VITALI |
| SOMMINISTRAZIONE QUESTIONARIO |
| VALUTAZIONE PARAMETRI VITALI E ANNOTAZIONE NEL DIARIO CLINICO |
| VISITA FINALE, DIMISSIONE E ANNOTAZIONE NEL DIARIO CLINICO |
| VISITA ONCOLOGICA E ANNOTAZIONE NEL DIARIO CLINICO |
| **2° FOLLOW-UP TRIMESTRALI SUCCESSIVI** |
| INSERIMENTO DATI NEL DATABASE |
| PROGRAMMAZIONE ATTIVITÀ DIAGNOSTICA (ESAMI E VISITE SPECIALISTICHE) |
| RACCOLTA E ANNOTAZIONE PARAMETRI VITALI |
| SOMMINISTRAZIONE QUESTIONARIO |
| VALUTAZIONE PARAMETRI VITALI E ANNOTAZIONE NEL DIARIO CLINICO |
| VISITA FINALE, DIMISSIONE E ANNOTAZIONE NEL DIARIO CLINICO |
| VISITA ONCOLOGICA E ANNOTAZIONE NEL DIARIO CLINICO |
| **1° MONITORAGGIO DELLO SPONSOR** |
| VALUTAZIONE ATTIVITA' SVOLTE |
| **Totale complessivo** |

| **ACTIVITY POOL AND MAIN ACTIVITIES** |
| --- |
|  |
| **Etichette di riga** |
| **PRE STUDY** |
| REVIEW THE ADEQUACY OF THE SITE |
| SITE INITIATION VISIT-CARDIOLOGY VISIT |
| SITE INITIATION VISIT-LABORATORY VISIT |
| SITE INITIATION VISIT-PATHOLOGY VISIT |
| SITE INITIATION VISIT-PHARMACY VISIT |
| SITE INITIATION VISIT-PROPOSAL AND SIGN OF STUDY PROTOCOL |
| SITE INITIATION VISIT-RADIODIAGNOSTIC 1 SERVICE VISIT |
| SITE INITIATION VISIT-RADIODIAGNOSTIC 2 SERVICE VISIT |
| **SCREENING** |
| BLOOD SAMPLE AND LABORATORY TESTS |
| CARDIOLOGIC TEST INTERPRETATION AND DOCUMENTAZION IN HEALTH RECORD |
| CARDIOLOGICAL VISIT AND ELECTROCARDIOGRAM |
| COMMUNICATION TO THE PATIENT OF THE TESTS DATE |
| DATA ENTRY IN THE SPONSOR SITE |
| EXTERNAL TEST INTERPRETATION |
| ISTOLOGICAL EXAM |
| LABORATORY TEST INTERPRETATION AND DOCUMENTATION IN HEALTH RECORD |
| ONCOLOGICAL VISIT AND DOCUMENTATION IN HEALTH RECORD |
| OTHER EXAMS (EVENTUALLY PROVIDED BY THE PROTOCOL) |
| OTHERS TEST INTERPRETATION AND DOCUMENTATION IN HEALTH RECORD |
| PATIENT ASSESSMENT |
| PLANNING OF DIAGNOSTIC ACTIVITY (TESTS AND MEDICAL EXAMINATIONS) |
| RADIOLOGIC TEST INTERPRETATION AND DOCUMENTAZION IN HEALTH RECORD |
| RADIOLOGICAL EXAMINATION |
| SHIPPING ISTOLOGICAL SAMPLES TO THE SPONSOR |
| VITAL SIGNS EVALUATION AND DOCUMENTATION IN HEALTH RECORD |
| VITAL SIGNS MEASUREMENT AND DOCUMENTATION IN HEALTH RECORD |
| **AUDIT** |
| IF PLANNED IN THE STUDY |
| **ENROLLMENT** |
| IVRS CALL |
| MEDICAL RECORD SET-UP |
| PATIENT IDENTIFICATION |
| PATIENT TELEPHONE CALL |
| SIGNATURE INFORMED CONSENT |
| **RANDOMIZATION** |
| IVRS CALL |
| **CYCLE 1** |
| BLOOD SAMPLE AND LABORATORY TESTS |
| CLINICAL TRIAL PATIENT FEEDBACK QUESTIONNAIRE |
| DATA ENTRY IN THE SPONSOR SITE |
| DRUG ADMINISTRATION |
| DRUG RECEPTION AND IVRS CALL |
| EXPERIMENTAL DRUG PREPARATION |
| EXTERNAL TEST INTERPRETATION |
| LABORATORY TEST INTERPRETATION AND DOCUMENTATION IN HEALTH RECORD |
| ONCOLOGICAL VISIT AND DOCUMENTATION IN HEALTH RECORD |
| ONCOLOGICAL VISIT, DISCHARGE AND DOCUMENTATION IN HEALTH RECORD |
| RECEIVING, MANAGEMENT AND SENDING EXPERIMENTAL DRUG |
| VITAL SIGNS EVALUATION AND DOCUMENTATION IN HEALTH RECORD |
| VITAL SIGNS MEASUREMENT AND DOCUMENTATION IN HEALTH RECORD |
| **CYCLE 2** |
| BLOOD SAMPLE AND LABORATORY TESTS |
| CLINICAL TRIAL PATIENT FEEDBACK QUESTIONNAIRE |
| DATA ENTRY IN THE SPONSOR SITE |
| DRUG ADMINISTRATION |
| DRUG RECEPTION AND IVRS CALL |
| EXPERIMENTAL DRUG PREPARATION |
| LABORATORY TEST INTERPRETATION AND DOCUMENTATION IN HEALTH RECORD |
| ONCOLOGICAL VISIT AND DOCUMENTATION IN HEALTH RECORD |
| ONCOLOGICAL VISIT, DISCHARGE AND DOCUMENTATION IN HEALTH RECORD |
| RECEIVING, MANAGEMENT AND SENDING EXPERIMENTAL DRUG |
| THERAPY DAY ORGANIZATION AND PATIENT COMMUNICATION |
| VITAL SIGNS EVALUATION AND DOCUMENTATION IN HEALTH RECORD |
| VITAL SIGNS MEASUREMENT AND DOCUMENTATION IN HEALTH RECORD |
| **CYCLE 3** |
| BLOOD SAMPLE AND LABORATORY TESTS |
| CLINICAL TRIAL PATIENT FEEDBACK QUESTIONNAIRE |
| DATA ENTRY IN THE SPONSOR SITE |
| DRUG ADMINISTRATION |
| DRUG RECEPTION AND IVRS CALL |
| EXPERIMENTAL DRUG PREPARATION |
| LABORATORY TEST INTERPRETATION AND DOCUMENTATION IN HEALTH RECORD |
| ONCOLOGICAL VISIT AND DOCUMENTATION IN HEALTH RECORD |
| ONCOLOGICAL VISIT, DISCHARGE AND DOCUMENTATION IN HEALTH RECORD |
| RECEIVING, MANAGEMENT AND SENDING EXPERIMENTAL DRUG |
| THERAPY DAY ORGANIZATION AND PATIENT COMMUNICATION |
| VITAL SIGNS EVALUATION AND DOCUMENTATION IN HEALTH RECORD |
| VITAL SIGNS MEASUREMENT AND DOCUMENTATION IN HEALTH RECORD |
| **1st RESTAGING** |
| DATA ENTRY IN THE SPONSOR SITE |
| PLANNING OF DIAGNOSTIC ACTIVITY (TESTS AND MEDICAL EXAMINATIONS) |
| RADIOLOGIC TEST INTERPRETATION AND DOCUMENTAZION IN HEALTH RECORD |
| RADIOLOGICAL EXAMINATION |
| (vuoto) |
| **CYCLE 4** |
| BLOOD SAMPLE AND LABORATORY TESTS |
| CLINICAL TRIAL PATIENT FEEDBACK QUESTIONNAIRE |
| DATA ENTRY IN THE SPONSOR SITE |
| DRUG ADMINISTRATION |
| DRUG RECEPTION AND IVRS CALL |
| EXPERIMENTAL DRUG PREPARATION |
| LABORATORY TEST INTERPRETATION AND DOCUMENTATION IN HEALTH RECORD |
| ONCOLOGICAL VISIT AND DOCUMENTATION IN HEALTH RECORD |
| ONCOLOGICAL VISIT, DISCHARGE AND DOCUMENTATION IN HEALTH RECORD |
| RECEIVING, MANAGEMENT AND SENDING EXPERIMENTAL DRUG |
| THERAPY DAY ORGANIZATION AND PATIENT COMMUNICATION |
| VITAL SIGNS EVALUATION AND DOCUMENTATION IN HEALTH RECORD |
| VITAL SIGNS MEASUREMENT AND DOCUMENTATION IN HEALTH RECORD |
| **CYCLE 5** |
| BLOOD SAMPLE AND LABORATORY TESTS |
| CLINICAL TRIAL PATIENT FEEDBACK QUESTIONNAIRE |
| DATA ENTRY IN THE SPONSOR SITE |
| DRUG ADMINISTRATION |
| DRUG RECEPTION AND IVRS CALL |
| EXPERIMENTAL DRUG PREPARATION |
| LABORATORY TEST INTERPRETATION AND DOCUMENTATION IN HEALTH RECORD |
| ONCOLOGICAL VISIT AND DOCUMENTATION IN HEALTH RECORD |
| ONCOLOGICAL VISIT, DISCHARGE AND DOCUMENTATION IN HEALTH RECORD |
| RECEIVING, MANAGEMENT AND SENDING EXPERIMENTAL DRUG |
| THERAPY DAY ORGANIZATION AND PATIENT COMMUNICATION |
| VITAL SIGNS EVALUATION AND DOCUMENTATION IN HEALTH RECORD |
| VITAL SIGNS MEASUREMENT AND DOCUMENTATION IN HEALTH RECORD |
| **CYCLE 6** |
| BLOOD SAMPLE AND LABORATORY TESTS |
| CLINICAL TRIAL PATIENT FEEDBACK QUESTIONNAIRE |
| DATA ENTRY IN THE SPONSOR SITE |
| DRUG ADMINISTRATION |
| DRUG RECEPTION AND IVRS CALL |
| EXPERIMENTAL DRUG PREPARATION |
| LABORATORY TEST INTERPRETATION AND DOCUMENTATION IN HEALTH RECORD |
| ONCOLOGICAL VISIT AND DOCUMENTATION IN HEALTH RECORD |
| ONCOLOGICAL VISIT, DISCHARGE AND DOCUMENTATION IN HEALTH RECORD |
| RECEIVING, MANAGEMENT AND SENDING EXPERIMENTAL DRUG |
| THERAPY DAY ORGANIZATION AND PATIENT COMMUNICATION |
| VITAL SIGNS EVALUATION AND DOCUMENTATION IN HEALTH RECORD |
| VITAL SIGNS MEASUREMENT AND DOCUMENTATION IN HEALTH RECORD |
| **2st RESTAGING** |
| DATA ENTRY IN THE SPONSOR SITE |
| PLANNING OF DIAGNOSTIC ACTIVITY (TESTS AND MEDICAL EXAMINATIONS) |
| RADIOLOGIC TEST INTERPRETATION AND DOCUMENTAZION IN HEALTH RECORD |
| RADIOLOGICAL EXAMINATION |
| (vuoto) |
| **CYCLE 7** |
| BLOOD SAMPLE AND LABORATORY TESTS |
| CLINICAL TRIAL PATIENT FEEDBACK QUESTIONNAIRE |
| DATA ENTRY IN THE SPONSOR SITE |
| DRUG ADMINISTRATION |
| DRUG RECEPTION AND IVRS CALL |
| EXPERIMENTAL DRUG PREPARATION |
| LABORATORY TEST INTERPRETATION AND DOCUMENTATION IN HEALTH RECORD |
| ONCOLOGICAL VISIT AND DOCUMENTATION IN HEALTH RECORD |
| ONCOLOGICAL VISIT, DISCHARGE AND DOCUMENTATION IN HEALTH RECORD |
| RECEIVING, MANAGEMENT AND SENDING EXPERIMENTAL DRUG |
| THERAPY DAY ORGANIZATION AND PATIENT COMMUNICATION |
| VITAL SIGNS EVALUATION AND DOCUMENTATION IN HEALTH RECORD |
| VITAL SIGNS MEASUREMENT AND DOCUMENTATION IN HEALTH RECORD |
| **CYCLE 8** |
| BLOOD SAMPLE AND LABORATORY TESTS |
| CLINICAL TRIAL PATIENT FEEDBACK QUESTIONNAIRE |
| DATA ENTRY IN THE SPONSOR SITE |
| DRUG ADMINISTRATION |
| DRUG RECEPTION AND IVRS CALL |
| EXPERIMENTAL DRUG PREPARATION |
| LABORATORY TEST INTERPRETATION AND DOCUMENTATION IN HEALTH RECORD |
| ONCOLOGICAL VISIT AND DOCUMENTATION IN HEALTH RECORD |
| ONCOLOGICAL VISIT, DISCHARGE AND DOCUMENTATION IN HEALTH RECORD |
| RECEIVING, MANAGEMENT AND SENDING EXPERIMENTAL DRUG |
| THERAPY DAY ORGANIZATION AND PATIENT COMMUNICATION |
| VITAL SIGNS EVALUATION AND DOCUMENTATION IN HEALTH RECORD |
| VITAL SIGNS MEASUREMENT AND DOCUMENTATION IN HEALTH RECORD |
| **CYCLE 9** |
| BLOOD SAMPLE AND LABORATORY TESTS |
| CLINICAL TRIAL PATIENT FEEDBACK QUESTIONNAIRE |
| DATA ENTRY IN THE SPONSOR SITE |
| DRUG ADMINISTRATION |
| DRUG RECEPTION AND IVRS CALL |
| EXPERIMENTAL DRUG PREPARATION |
| LABORATORY TEST INTERPRETATION AND DOCUMENTATION IN HEALTH RECORD |
| ONCOLOGICAL VISIT AND DOCUMENTATION IN HEALTH RECORD |
| ONCOLOGICAL VISIT, DISCHARGE AND DOCUMENTATION IN HEALTH RECORD |
| RECEIVING, MANAGEMENT AND SENDING EXPERIMENTAL DRUG |
| THERAPY DAY ORGANIZATION AND PATIENT COMMUNICATION |
| VITAL SIGNS EVALUATION AND DOCUMENTATION IN HEALTH RECORD |
| VITAL SIGNS MEASUREMENT AND DOCUMENTATION IN HEALTH RECORD |
| **3st RESTAGING** |
| DATA ENTRY IN THE SPONSOR SITE |
| PLANNING OF DIAGNOSTIC ACTIVITY (TESTS AND MEDICAL EXAMINATIONS) |
| RADIOLOGIC TEST INTERPRETATION AND DOCUMENTAZION IN HEALTH RECORD |
| RADIOLOGICAL EXAMINATION |
| (vuoto) |
| **CYCLE 10** |
| BLOOD SAMPLE AND LABORATORY TESTS |
| CLINICAL TRIAL PATIENT FEEDBACK QUESTIONNAIRE |
| DATA ENTRY IN THE SPONSOR SITE |
| DRUG ADMINISTRATION |
| DRUG RECEPTION AND IVRS CALL |
| EXPERIMENTAL DRUG PREPARATION |
| LABORATORY TEST INTERPRETATION AND DOCUMENTATION IN HEALTH RECORD |
| ONCOLOGICAL VISIT AND DOCUMENTATION IN HEALTH RECORD |
| ONCOLOGICAL VISIT, DISCHARGE AND DOCUMENTATION IN HEALTH RECORD |
| RECEIVING, MANAGEMENT AND SENDING EXPERIMENTAL DRUG |
| THERAPY DAY ORGANIZATION AND PATIENT COMMUNICATION |
| VITAL SIGNS EVALUATION AND DOCUMENTATION IN HEALTH RECORD |
| VITAL SIGNS MEASUREMENT AND DOCUMENTATION IN HEALTH RECORD |
| **CYCLE 11** |
| BLOOD SAMPLE AND LABORATORY TESTS |
| CLINICAL TRIAL PATIENT FEEDBACK QUESTIONNAIRE |
| DATA ENTRY IN THE SPONSOR SITE |
| DRUG ADMINISTRATION |
| DRUG RECEPTION AND IVRS CALL |
| EXPERIMENTAL DRUG PREPARATION |
| LABORATORY TEST INTERPRETATION AND DOCUMENTATION IN HEALTH RECORD |
| ONCOLOGICAL VISIT AND DOCUMENTATION IN HEALTH RECORD |
| ONCOLOGICAL VISIT, DISCHARGE AND DOCUMENTATION IN HEALTH RECORD |
| RECEIVING, MANAGEMENT AND SENDING EXPERIMENTAL DRUG |
| THERAPY DAY ORGANIZATION AND PATIENT COMMUNICATION |
| VITAL SIGNS EVALUATION AND DOCUMENTATION IN HEALTH RECORD |
| VITAL SIGNS MEASUREMENT AND DOCUMENTATION IN HEALTH RECORD |
| **CYCLE 12** |
| BLOOD SAMPLE AND LABORATORY TESTS |
| CLINICAL TRIAL PATIENT FEEDBACK QUESTIONNAIRE |
| DATA ENTRY IN THE SPONSOR SITE |
| DRUG ADMINISTRATION |
| DRUG RECEPTION AND IVRS CALL |
| EXPERIMENTAL DRUG PREPARATION |
| LABORATORY TEST INTERPRETATION AND DOCUMENTATION IN HEALTH RECORD |
| ONCOLOGICAL VISIT AND DOCUMENTATION IN HEALTH RECORD |
| ONCOLOGICAL VISIT, DISCHARGE AND DOCUMENTATION IN HEALTH RECORD |
| RECEIVING, MANAGEMENT AND SENDING EXPERIMENTAL DRUG |
| THERAPY DAY ORGANIZATION AND PATIENT COMMUNICATION |
| VITAL SIGNS EVALUATION AND DOCUMENTATION IN HEALTH RECORD |
| VITAL SIGNS MEASUREMENT AND DOCUMENTATION IN HEALTH RECORD |
| **4st RESTAGING** |
| DATA ENTRY IN THE SPONSOR SITE |
| PLANNING OF DIAGNOSTIC ACTIVITY (TESTS AND MEDICAL EXAMINATIONS) |
| RADIOLOGIC TEST INTERPRETATION AND DOCUMENTAZION IN HEALTH RECORD |
| RADIOLOGICAL EXAMINATION |
| (vuoto) |
| **1st TRIAL MONITORING** |
| OVERSEEING THE PROGRESS OF THE CLINICAL TRIAL |
| **EXIT CANCER VISIT** |
| BLOOD SAMPLE AND LABORATORY TESTS |
| CLINICAL TRIAL PATIENT FEEDBACK QUESTIONNAIRE |
| DATA ENTRY IN THE SPONSOR SITE |
| IVRS CALL FOR EXIT PATIENT |
| LABORATORY TEST INTERPRETATION AND DOCUMENTATION IN HEALTH RECORD |
| ONCOLOGICAL VISIT AND DOCUMENTATION IN HEALTH RECORD |
| ONCOLOGICAL VISIT, DISCHARGE AND DOCUMENTATION IN HEALTH RECORD |
| PLANNING OF DIAGNOSTIC ACTIVITY (TESTS AND MEDICAL EXAMINATIONS) |
| VITAL SIGNS EVALUATION AND DOCUMENTATION IN HEALTH RECORD |
| VITAL SIGNS MEASUREMENT AND DOCUMENTATION IN HEALTH RECORD |
| **35-DAY FOLLOW-UP** |
| BLOOD SAMPLE AND LABORATORY TESTS |
| CLINICAL TRIAL PATIENT FEEDBACK QUESTIONNAIRE |
| DATA ENTRY IN THE SPONSOR SITE |
| LABORATORY TEST INTERPRETATION AND DOCUMENTATION IN HEALTH RECORD |
| ONCOLOGICAL VISIT AND DOCUMENTATION IN HEALTH RECORD |
| ONCOLOGICAL VISIT, DISCHARGE AND DOCUMENTATION IN HEALTH RECORD |
| PLANNING OF DIAGNOSTIC ACTIVITY (TESTS AND MEDICAL EXAMINATIONS) |
| VITAL SIGNS EVALUATION AND DOCUMENTATION IN HEALTH RECORD |
| VITAL SIGNS MEASUREMENT AND DOCUMENTATION IN HEALTH RECORD |
| **90-DAY FOLLOW-UP** |
| BLOOD SAMPLE AND LABORATORY TESTS |
| CLINICAL TRIAL PATIENT FEEDBACK QUESTIONNAIRE |
| DATA ENTRY IN THE SPONSOR SITE |
| LABORATORY TEST INTERPRETATION AND DOCUMENTATION IN HEALTH RECORD |
| ONCOLOGICAL VISIT AND DOCUMENTATION IN HEALTH RECORD |
| ONCOLOGICAL VISIT, DISCHARGE AND DOCUMENTATION IN HEALTH RECORD |
| PLANNING OF DIAGNOSTIC ACTIVITY (TESTS AND MEDICAL EXAMINATIONS) |
| VITAL SIGNS EVALUATION AND DOCUMENTATION IN HEALTH RECORD |
| VITAL SIGNS MEASUREMENT AND DOCUMENTATION IN HEALTH RECORD |
| **1st SURVIVAL FOLLOW-UP** |
| CLINICAL TRIAL PATIENT FEEDBACK QUESTIONNAIRE |
| DATA ENTRY IN THE SPONSOR SITE |
| ONCOLOGICAL VISIT AND DOCUMENTATION IN HEALTH RECORD |
| ONCOLOGICAL VISIT, DISCHARGE AND DOCUMENTATION IN HEALTH RECORD |
| PLANNING OF DIAGNOSTIC ACTIVITY (TESTS AND MEDICAL EXAMINATIONS) |
| VITAL SIGNS EVALUATION AND DOCUMENTATION IN HEALTH RECORD |
| VITAL SIGNS MEASUREMENT AND DOCUMENTATION IN HEALTH RECORD |
| **2st SURVIVAL FOLLOW-UP** |
| CLINICAL TRIAL PATIENT FEEDBACK QUESTIONNAIRE |
| DATA ENTRY IN THE SPONSOR SITE |
| ONCOLOGICAL VISIT AND DOCUMENTATION IN HEALTH RECORD |
| ONCOLOGICAL VISIT, DISCHARGE AND DOCUMENTATION IN HEALTH RECORD |
| PLANNING OF DIAGNOSTIC ACTIVITY (TESTS AND MEDICAL EXAMINATIONS) |
| VITAL SIGNS EVALUATION AND DOCUMENTATION IN HEALTH RECORD |
| VITAL SIGNS MEASUREMENT AND DOCUMENTATION IN HEALTH RECORD |
| **ADMINISTRATIVE ACTIVITY** |
| ADMINISTRATIVE ACTIVITY |
| INVOICING FOR TRIAL PAYMENTS |
| **Totale complessivo** |
|  |

1. The subsequent step was to define the resources used for each activity identified for each trial, calculating the time required to perform all the planned activities, the name and the role of the employees who performed them (e.g., doctors, nurses, pharmacists, laboratory technicians, x-ray technicians, study coordinators, etc.) for all unit involved in the studies.

1. The cost of the time required to perform an activity was estimated by using labour hourly rate of each employer, while the cost of diagnostic tests and other procedures was estimated by using public health reimbursement rates. All other costs, not directly related to a specific activity, were included in an overhead cost category estimated at 20% of the cost of all activities performed
2. We then built an Expense-Activity-Link Matrix (EAL-matrix) for each clinical trial (reported as table 2 of web appendix).
3. Finally, since the clinical trials were structured differently, a bridging table was developed by grouping all the primary activities performed in six main categories, as follows: a) pre-study activities, including “Pre-Study Site Visit, Enrollment and Screening Phases”; b) treatment, including “Cycles, Restaging and Exit Cancer Visit”; c) trial monitoring; d) follow-up; e) audit (generally only one for each study); f) administrative activities (reported as table 3 of web-appendix).
