# Supplementary material for: Costs of clinical trials with anticancer biological agents in an Oncologic Italian Cancer Center using the activity-based costing methodology
Source: PLoS One. 2019 Jan 8;14(1):e0210330. doi: 10.1371/journal.pone.0210330 (PMC6324822; doi:10.1371/journal.pone.0210330)
Supplement: Supplementary file 16 [file pone.0210330.s002.docx]

S2 Table. Expense-Activity-Link Matrix (EAL-matrix)

FIRST STAGE COST ASSIGNMENT: ALLOCATING RESOURCES COST TO ACTIVITY POOLS

	LOCAL CLINICAL TRIAL UNIT COSTS		INTERMEDIATE SERVICES COSTS																
ACTIVITY POOLS	OVARIAN MEDICAL ONCOLOGY		RADIODIAGNOSTIC 1		RADIODIAGNOSTIC 2		NUCLEAR MEDICINE		PHARMACY		CLINICAL LABORATORY		PATHOLOGICAL ANATOMY		CARDIOLOGY		MOLECULAR BIOLOGY		ACTIVITY COST POOLS
	STAFF	TEST	STAFF	TEST	STAFF	TEST	STAFF	TEST	STAFF	TEST	STAFF	TEST	STAFF	TEST	STAFF	TEST	STAFF	TEST	
PRE STUDY SITE VISITS																			0,00
ENROLLMENT																			0,00
SCREENING																			0,00
RANDOMIZATION																			0,00
CYCLE 1 DAY 1																			0,00
CYCLE 1 DAY 15																			0,00
1st TRIAL MONITORING																			0,00
CYCLE 2 DAY 1																			0,00
CYCLE 2 DAY 15																			0,00
2st TRIAL MONITORING																			0,00
CYCLE 3 DAY 1																			0,00
1st RESTAGING																			0,00
3st TRIAL MONITORING																			0,00
CYCLE 4 DAY 1																			0,00
4st TRIAL MONITORING																			0,00
CYCLE 5 DAY 1																			0,00
5st TRIAL MONITORING																			0,00
CYCLE 6 DAY 1																			0,00
1st RESTAGING																			0,00
6st TRIAL MONITORING																			0,00
EXIT CANCER VISIT																			0,00
30-DAY FOLLOW-UP																			0,00
90-DAY PHONE FOLLOW-UP																			0,00
190-DAY PHONE FOLLOW-UP																			0,00
280-DAY PHONE FOLLOW-UP																			0,00
370-DAY PHONE FOLLOW-UP																			0,00
460-DAY PHONE FOLLOW-UP																			0,00
550-DAY PHONE FOLLOW-UP																			0,00
640-DAY PHONE FOLLOW-UP																			0,00
730-DAY PHONE FOLLOW-UP																			0,00
AUDIT																			0,00
ADMINISTRATIVE ACTIVITY																			0,00
**ESTIMATED COST OF RESOURCES**	**0,00**	**0,00**	**0,00**	**0,00**	**0,00**	**0,00**	**0,00**	**0,00**	**0,00**	**0,00**	**0,00**	**0,00**	**0,00**	**0,00**	**0,00**	**0,00**	**0,00**	**0,00**	**0,00**
**ESTIMATED COST OF OVERHEAD (20%)**																			**0,00**
**ESTIMATED TOTAL COST or PER PATIENT COST**																			**0,00**
STAFF COSTS																			**0,00**
TEST COSTS																			**0,00**
INCIDENCE STAFF COSTS ON ESTIMATED COST OF RESOURCES																			**0,00**
INCIDENCE TEST COSTS ON ESTIMATED COST OF RESOURCES																			**0,00**

ESTIMATED COST OF ACTIVITIES PERFORMED
